# Production and characterization of mechanically-enzymatically treated suspensions of the food by-product pea hull

**DOI:** 10.3389/fnut.2026.1852207

**Published:** 2026-07-10

**Authors:** Rebekka Elke Schmidt, Veronika Kurz, Verena Haitz, Rocío Morales-Medina, Jan Steffan, Judith Keller, Stephan Drusch, Mirko Bunzel

**Affiliations:** 1 Department of Food Chemistry and Phytochemistry, Institute of Applied Biosciences, Karlsruhe Institute of Technology (KIT), Karlsruhe, Germany; 2 Department of Food Technology and Food Material Science, Institute of Food Technology and Food Chemistry, Technische Universität Berlin (TUB), Berlin, Germany

**Keywords:** cell wall polysaccharides, dietary fiber, dynamic high-pressure microfluidization, enzymatic hydrolysis, food by-product, upcycling

## Abstract

Given the potential health benefits of dietary fiber, incorporating high-fiber by-products such as pea hulls into food products is both sustainable and desirable. However, incorporation into semi-solid or low viscous food products can be challenging due to limited fiber functionality, which is partly attributable to its low solubility. Here, a combined mechanical-enzymatic treatment of pea hulls was performed with the aim of obtaining low viscous, physically stable, oligosaccharide-rich fiber suspensions. The pea hull consisted mainly of cellulose next to (glucurono)xylans, pectic polysaccharides, especially linear arabinans and unesterified homogalacturonans, and xyloglucans. Mechanical-enzymatic treatment of pea hulls was performed using microfluidization (1,750 bar, 3 or 5 runs), and commercial arabinanase and cellulase (50 °C, 30 min) were used for the enzymatic treatment. The chemical-structural and the technofunctional properties of the treated pea hulls were determined. Both mechanically-enzymatically treated pea hulls showed comparable fiber contents (73%−75%) as well as polysaccharide composition and structures. Both treatments resulted in a reduction in particle size, though with different particle size distributions, and improved functional properties of pea hull suspensions. High mechanical intensity was crucial for physical stability of the suspensions and enzymes for partial degradation of polysaccharides. Contrary to our expectations, this was not accompanied by extensive solubilization of insoluble cell wall polysaccharides, which is likely due to the high proportion of crystalline cellulose. Nevertheless, the treated pea hulls showed an improved hydration capacity, and limited amounts of potentially prebiotic di- and oligosaccharides were also released. Overall, the treatments resulted in high-fiber ingredients with small particle sizes (D_90_ between 94 and 121 μm) that exhibited improved hydration and sedimentation properties, as well as higher levels of potentially prebiotic carbohydrates, which appear suitable for incorporation into semi-solid and/or solid food products.

## 1 Introduction

Dietary fiber represents a heterogeneous group of various components, which mainly originate from plant cell walls. Dietary fiber can be divided into water-insoluble (IDF), soluble (SDF, ethanol-insoluble), and low-molecular weight soluble dietary fiber (LMWSDF) (1). Dominant components are often cell wall polysaccharides such as cellulose, hemicelluloses, and pectins (2). For these polysaccharides and related oligosaccharides many different positive nutritional effects are postulated. However, depending on their structure, cell wall polysaccharides behave differently in the gastrointestinal tract (3). Increased intake of dietary fiber can reduce blood cholesterol levels as well as the risk of colorectal cancer (2, 4). Moreover, dietary fiber stimulates the growth of and potentially shapes the gut microbiome (2–5). It has been described that the prebiotic potential of pectic oligosaccharides is higher as compared to pectins (6). Also, the degree of polymerization (DP) of the oligosaccharides has an impact on their prebiotic potential (7).

The European Food Safety Authority (EFSA) recommended a daily intake of 25 g of dietary fiber for adults (8), while the National Academy of Medicine recommended up to 38 g dietary fiber per day (9). An increased intake of dietary fiber can be achieved by enriching various foods with dietary fiber. In the vegetable processing industry, by-products include, for example, hulls (botanically, in case of peas, the seed coat) that are removed before starch and/or proteins are extracted from peas. These are usually rich in dietary fiber and can be used for enriching low-fiber food products, which does not only offer nutritional benefits but is also sustainable. Depending on fiber polysaccharide structures, dietary fiber is insoluble, creates viscosity, or even forms gels. Consequently, the addition of dietary fiber to semi-solid food products such as yogurts or low-viscous products such as beverages without undesired textural changes is only possible to a limited extent.

Dynamic high-pressure microfluidization is a high-pressure homogenization process that is characterized by low nutrient loss, low temperatures, continuous operation, and short process times (10). It can be used to improve functional properties of, for example, IDF of peach, oat, or pea (hull) fiber as previously described (11–13). In newer approaches with peach pomace and pea hull the high-pressure treatment is combined with enzymatic hydrolysis (14, 15). Thereby, the dietary fiber structures are thought to be modified by mechanical loosening of the cell wall structures and enzymatic degradation of polysaccharides. Depending on the fiber source, various cell wall polysaccharide degrading enzymes are suitable for enzymatic hydrolysis.

In this study a mechanical pre-treatment with subsequent enzymatic hydrolysis was selected for targeted functionalization of pea hulls. The process was aimed to be optimized to get physically stable pea hull suspensions of low viscosity that contain oligosaccharides, thus including a reduction of IDF contents by conversion to SDF and/or LMWSDF. In addition, we aimed to track these processes through detailed chemical analyses of the various fiber fractions in order to gain a deeper understanding of the processing steps.

## 2 Materials and methods

### 2.1 Materials

Dried and milled pea hull (Empet E5 B10, particle size: 500 μm) was kindly provided by Emsland-Stärke GmbH (Emlichheim, Germany), and citrus pectins Pectin Classic CU201 [high degree of methylation (DM)] and Pectin Classic CU901 (low DM) were kindly made available by Herbstreith & Fox (Neuenbürg, Germany). Commercially available arabinanase, cellulase, polygalacturonanase, and xylanase preparations were used to enzymatically hydrolyze pea hull. The enzymes α-amylase (thermostable, E 3.2.1.1, from *Bacillus licheniformis*), amyloglucosidase (EC 3.2.1.3, from *Aspergillus niger*), *endo*-arabinanase (EC 3.2.1.89, from *Aspergillus niger*), *endo*-galactanase (EC 3.2.1.99 from *Aspergillus niger*), protease (EC 3.4.21.14, from *Bacillus licheniformis*), and *endo*-xyloglucanase (EC 3.2.1.151 from *Paenibacillus* sp.) were purchased from Megazyme (Bray, Ireland), and the enzymes α-amylase (Termamyl 120 L, E 3.2.1.1, from *Bacillus licheniformis*), amyloglucosidase (EC 3.2.1.3, from *Aspergillus niger*), invertase (EC 3.2.1.26, from *Saccharomyces cerevisiae*), protease (EC 3.4.21.62, from *Bacillus licheniformis*) were from Sigma Aldrich (Schnelldorf, Germany). Arabino- (DP = 2–6), cello- (DP = 3–6), galacturonic acid (DP = 2–4), malto- (DP = 2–5), and xylooligosaccharides (DP = 2–6) as well as arabinan (sugar beet), verbascose, and ion exchange resins [Amberlite FFA53 (OH-), Ambersep 200 (H+)] were purchased from Megazyme. All other carbohydrate standards (arabinose, cellobiose, dextran standards (5, 12, 50, 150, 670 kDa), fructose, fucose, galactose, galactobiose, galacturonic acid, glucose, 2-deoxy-glucose, glucuronic acid, isomaltotriose, mannose, melezitose, raffinose, rhamnose, stacchyose, sucrose, xylan [(beechwood), xylose] as well as glycerol and other chemicals were purchased from Alfa Aesar GmbH & Co KG (Karlsruhe, Germany), Carl Roth GmbH & Co. KG (Karlsruhe, Germany), Deutero GmbH (Kastellaun, Germany), Fluka Chemie AG (Buchs, Switzerland), Merck KGaA (Darmstadt, Germany), Sigma Aldrich, Supelco Analytical (Bellefonte, USA), or VWR Chemicals (Radnor, PA, USA). Chemicals were usually of analytical grade. Partially branched arabino- (DP = 2–7) and (arabino-) galactooligosaccharides (DP = 2–4) were previously isolated and characterized as described by Wefers and Bunzel (16).

### 2.2 Production of mechanically(-enzymatically) treated pea hull

#### 2.2.1 Characterization of enzymes

Enzyme preparations (arabinanase, cellulase, polygalacturonanase, xylanase) were analyzed regarding their side-activities, optimum temperature, and heat inactivation. To determine whether potential side-activities affect polysaccharides that are relevant for pea hulls, materials representing these polysaccharides (arabinan, homogalacturonan-rich pectins, cellulose-rich pea hull, xylan) were hydrolyzed with each enzyme preparation for 60 min at 50 °C. Optimum temperature of each enzyme preparation was identified by hydrolysis of the targeted polysaccharide at 30, 40, 50, or 60 °C for 60 min. Inactivation of the enzymes was carried out at 95 °C for 10 min. To verify successful inactivation of the enzymes, solutions of each enzyme were heated up to 80 or 95 °C, respectively, for 10 min. Then the targeted polysaccharide of the enzyme was added and incubated at 50 °C for 60 min. Release of mono-, di- and oligosaccharides was monitored by analysis with high-pressure anion exchange chromatography (HPAEC) with pulsed amperometric detection (PAD) and parallel detection with mass spectrometry (MS) as described later.

#### 2.2.2 Dynamic high-pressure microfluidization

The raw material was ground sequentially employing an ultra-centrifugal miller ZM1 (Retsch Technology GmbH, Haan, Germany) equipped with a 12-tooth SS rotor (Retsch Technology GmbH, Haan, Germany). Pea hulls were ground employing sequentially 500 mm and 250 mm steel rings and then separated in a vibratory mechanical sieve (VIBRO, Retsch Technology GmbH, Haan, Germany) for 20 min and 80% amplitude, retaining the fraction of particle size below 140 μm for the subsequent steps. The milling-sieving steps were repeated for several cycles until the residual mass of particles bigger than 140 μm represented less than 5 wt% of the initial mass. The milled and sieved particles (particle size: ≤ 140 μm) were dispersed in distilled water (1 wt%), and pre-shared in an ULTRA-TURRAX T25 digital (IKA-Werke GmbH & Co. KG, Germany) for 2 min at 16.000 rpm.

The following dynamic high-pressure microfluidization was performed with an LM20 microfluidizer (Microfluidics Co., MA, USA) equipped with two exchangeable Z-type interaction chambers with inner channel diameters of 200 and 100 μm (H30z 200μ Ceramic S#17403 and H10z 100μ Diamond S#17406, Microfluidics Co). As pre-treatment, the first run with 1,750 bar was only done with the bigger chamber (200 μm) to prevent blockages in the smaller chamber (100 μm). Then the smaller chamber was connected in series behind the bigger chamber, and microfluidization was performed for another 1, 3, or 5 runs with 1,750 bar (PH-m-1/3/5). After every run, the suspension was cooled down to 30 °C in an ice bath. The microfluidized pea hull suspensions were directly enzymatically treated.

#### 2.2.3 Enzymatic hydrolysis

The aim of the enzymatic hydrolysis was to reduce the amount of IDF and to increase the amounts of SDF and LMWSDF, while keeping the contents of free mono- and disaccharides low. For optimization of the enzymatic hydrolysis, arabinanase, cellulase, polygalacturonanase, and xylanase were used. These enzymes were chosen based on the composition of the pea hull non-starch polysaccharides. Enzymes were added alone and in various mixtures and concentrations to the three mechanically treated pea hulls (PH-m-1/3/5). Incubations were performed at 50 °C for 10, 30, or 60 min, respectively. Samples were monitored by determining free mono- and disaccharides and by analyzing the molecular weight distribution of the water-soluble compounds (methods described in later chapters) to specify the best conditions of enzymatic hydrolysis.

Based on the preliminary screening of the impact of the number of runs during microfluidization and the enzymatic treatment, two different process conditions were selected in which pea hull suspensions were subsequently mechanically and enzymatically treated (i.e., PH-me-3 and PH-me-5). In this case, mechanically treated pea hulls (PH-m-3 and PH-m-5) were produced at a larger scale (1 wt%, two batches of 800 g each) to obtain sufficient material for subsequent physical and chemical characterization. After microfluidization, the suspensions were heated to 50 °C in a thermostatic water bath under constant agitation (300 rpm). Then, a mixture of arabinanase and cellulase (each 10 μL/g pea hull each) was added to the suspensions, and it was incubated for 30 min at 50 °C while stirring at 300 rpm. Following incubation, the enzymes were immediately inactivated at 95 °C for 10 min. After cooling to 30 °C in an ice bath, freshly treated suspensions were used to determine physical and functional properties. For chemical characterization, aliquots were frozen (−20 °C), freeze-dried, and milled (particle size ≤ 200 μm).

### 2.3 Characterization of untreated and treated pea hulls

The two mechanically-enzymatically treated pea hulls (PH-me-3 and PH-me-5) as well as the untreated (PH-u, milled and sieved, particle size: < 140 μm) and the two mechanically treated pea hulls (PH-m-3 and PH-m-5) were characterized to identify modifications of the dietary fiber contents, carbohydrate structures, and changes in functionality caused by the mechanical(-enzymatic) treatment.

#### 2.3.1 Determination of ash and protein

Ash contents were determined gravimetrically after incineration of the pea hull, IDF, or SDF (500 °C, 5 h). Protein contents of the pea hull, IDF, or SDF were analyzed according to the Kjeldahl principle. The released ammonium was converted into ammonia by adding sodium hydroxide and quantified using an ammonia-sensitive electrode (17). The general conversion factor of 6.25 was used to estimate the protein contents (18).

#### 2.3.2 Determination of free monosaccharides, disaccharides, and oligosaccharides of the raffinose family

Mono- and disaccharides (glucose, fructose, sucrose) as well as oligosaccharides of the raffinose family (RFO; raffinose, stachyose, verbascose) are native compounds of pea hulls (19). During mechanical-enzymatic treatment of pea hulls the release of mono- and disaccharides by degradation of poly- and oligosaccharides was expected. Thus, contents of free mono- and disaccharides and RFO were analyzed by HPAEC-PAD (Dionex ICS-5000 system, Thermo Fisher Scientific, Dreieich, Germany) using an analytical CarboPac PA20 column (150 × 3 mm, 6.5 μm particle size, Thermo Fisher Scientific). Separation of aliquots (25 μL) was performed at 25 °C with a flow rate of 0.4 mL/min using ultra-pure water (A), 0.1 M sodium hydroxide (B), and 0.1 M sodium hydroxide with 0.5 mM sodium acetate (C) as eluents. Due to coelution four different gradient programs (Supplementary Tables S1–S4) were required for the determination of (1) monosaccharides, (2) RFO, (3) arabinobiose, cellobiose, and xylobiose, and (4) digalacturonic acid. Depending on the gradient either 2-deoxy-glucose (final concentration: 10 μM) (gradients 1 and 2), melezitose (final concentration: 10 μM) (gradient 3), or isomaltotriose (final concentration: 2 μM) (gradient 4) was added to each sample as internal standard for the chromatography. Quantification was performed for each saccharide using an external calibration.

#### 2.3.3 Determination and isolation of dietary fiber

Dietary fiber contents were determined separately by an enzymatic-gravimetric approach for IDF, SDF, and LMWSDF according to a combination of the AOAC-methods 985.29 (20), 2009.01 (21), and 2017.16 (22). In brief, starch and protein were digested sequentially using α-amylase (thermostable; exclusion of resistant starch), protease, and amyloglucosidase. IDF and, following precipitation in 78% ethanol, SDF were removed separately, washed, dried, and weighed. The amounts of IDF and SDF were corrected for residual protein and ash contents as described above. The ethanolic supernatant (LMWSDF-fraction) after removal of SDF was analyzed by high performance size-exclusion chromatography (HPSEC, Hitachi, Merck, Darmstadt, Germany) with refractive index detection (RID, Knauer, Berlin, Germany) using a TSKgel PW_XL_ guard column (40 mm × 6.0 mm, 12 μm particle size, Tosoh, Tokyo, Japan) and two size-exclusion columns in series (TSKgel G2500PW_XL_, 300 mm × 7.8 mm, 13 μm particle size, Tosoh). Ultra-pure water was used for isocratic separation of the desalted aqueous LMWSDF-fraction (50 μL) at 80 °C with a flow rate of 0.4 mL/min. The retention times of maltose and maltotriose were used to differentiate between LMWSDF and mono- and disaccharides. The content of LMWSDF was estimated by external calibration with glucose using glycerol as internal standard.

On a preparative scale, IDF and SDF were isolated as previously described by Bunzel et al. (23) being based on the principles of AOAC-method 985.29 (20), thus, including the removal of resistant starch. LMWSDF-fractions were isolated using a modification of the AOAC-methods 2009.01 (21) and 2017.16 (22) without using buffers. First, starch and maltooligosaccharides were enzymatically digested. Each sample (140 mg) was suspended in 9.8 mL of water, 28 μL of a solution of amyloglucosidase (9.5 mg/1 mL) was added, and the suspension was heated at 60 °C for 30 min. After centrifugation, IDF was removed. SDF was precipitated in 78 % ethanol and also removed by centrifugation. The LMWSDF-fractions (on a preparative scale defined as the ethanolic supernatants) were dried by rotary evaporation and diluted in 2.4 mL of water. Thus, differently from the analytical approach, preparative LMWSDF-fractions contain all components (oligo-, di-, and monosaccharides) that are soluble in 78 % ethanol.

#### 2.3.4 Monomer composition of polysaccharides

Monomer composition of IDF polysaccharides was determined after acidic hydrolysis following the principles of Saeman et al. (24). In brief, IDF was pre-hydrolyzed with 12 M sulfuric acid for 0.5 h on ice and for 2 h at room temperature. After dilution with water to 1.6 M sulfuric acid, hydrolysis was performed at 100 °C for 3 h. The cooled hydrolysate was filtered (PTFE, 0.45 μm), and the supernatant was neutralized with sodium hydroxide (4 M) and diluted with water.

Determination of the monomer composition of the oligo- and/or polysaccharides of IDF [in addition to the analysis following Saeman et al. (24) hydrolysis], SDF, and LMWSDF-fractions, respectively, was performed after acidic methanolysis combined with trifluoroacetic acid (TFA)-hydrolysis according to De Ruiter et al. (25). Briefly, samples were hydrolyzed with methanolic hydrochloric acid (1.25 M) for 16 h at 80 °C, dried by evaporation, and hydrolyzed with TFA (2 M) for 1 h at 121 °C. Samples were dried by evaporation and diluted with water. The monosaccharides liberated by acidic hydrolysis (sulfuric or methanolysis with TFA) were analyzed by HPAEC-PAD on a CarboPac PA20 column using the conditions described by Wefers and Bunzel (26).

#### 2.3.5 Glycosidic linkages within polysaccharides

The different glycosidic linkages in the polysaccharides of IDF and SDF were calculated on a molar distribution base, following the analysis of partially methylated alditol acetates (PMAA). Methylation analysis was based on the methods previously described by Nunes and Coimbra (27) and Gniechwitz et al. (28): methylation with methyl iodide in dimethyl sulfoxide and sodium hydroxide, extraction into dichloromethane, acidic hydrolysis with 2 M TFA (121 °C, 1.5 h), reduction with sodium borodeuteride-*d*_4_ in ammonia (2 M), and acetylation with acetic anhydride catalyzed by 1-methylimidazole. Before methylation, IDF and SDF were swollen overnight in dimethyl sulfoxide, and methylation was performed twice to reduce artifacts of incomplete methylation. Formed PMAA were analyzed by gas chromatography (GC)-MS (GC-2010 Plus and GCMS-QP2010 SE, Shimadzu, Kyoto, Japan) and GC with flame ionization detection (FID, GC-2010 Plus, Shimadzu) each on a DB-225MS column (30 m × 0.25 mm i.d., 0.25 μm film thickness, Agilent Technologies, Santa Clara, CA, USA) as described by Wefers and Bunzel (26). Amounts of PMAA were calculated according to the molar response factors from Sweet et al. (29).

#### 2.3.6 Fine structure of arabinans, (arabino-)galactans, and xyloglucans

**Figure 1:**
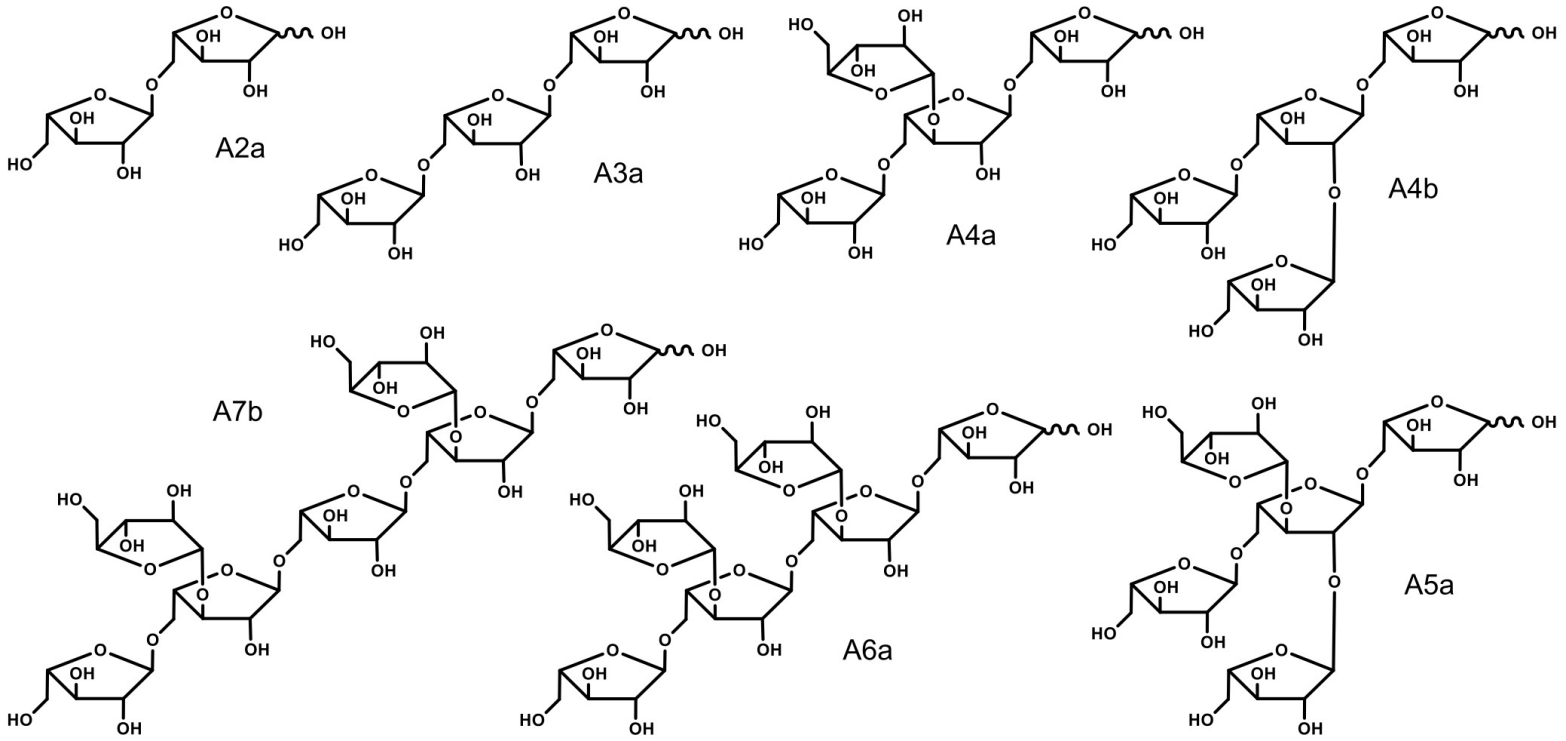
Structures of arabinooligosaccharides composed of α-l-arabinofuranoses and enzymatically released from pea hull; A2a, A4a, … = nomenclature according to Wefers and Bunzel (16).

For characterization of the fine structures of arabinans, (arabino-)galactans, and xyloglucans the in-house developed enzymatic-chromatographic-based profiling methods, published by Wefers and Bunzel (16) and Steck et al. (30), were used. In brief, *endo*-arabinanase, *endo*-galactanase, or *endo*-xyloglucanase, respectively, were added to IDF and SDF, and the suspensions were incubated (40 °C, 24 h). Concentrations of enzymes were adopted for IDF and SDF separately to reach a maximum degradation of arabinans, (arabino-)galactans, and xyloglucans, respectively. Released arabino-, (arabino-)galacto-, and xyloglucanoligosaccharides, respectively, were analyzed by HPAEC-PAD(-MS) (ISQ IC Mass Spectrometer, Thermo Fisher Scientific) on an analytical CarboPac PA200 column (250 × 3 mm, 5.5 μm particle size, Thermo Fisher Scientific) as previously described (16, 30). The structures of arabinooligosaccharides that were enzymatically released from pea hull are shown in Figure 1.

#### 2.3.7 Degree of esterification of polymer-bound uronic acids

The DM and degree of acetylation (DAc) were calculated by relating the contents of methanol or acetic acid released by alkaline hydrolysis to the separately determined content of polymer-bound galacturonic acids. Polymer-bound galacturonic acids was determined as described by Blumenkrantz and Asboe-Hansen (31). In brief, pre-treated IDF (12 M sulfuric acid) and SDF (aqueous solution) were heated (95 °C, 5 min) with sodium tetraborate (12.5 mM) in concentrated sulfuric acid. After cooling, a solution of 3-phenylphenol (0.15%) in sodium hydroxide (0.5%) was added, and the absorbance at 520 nm was measured in relation to a blank (sample without 3-phenylphenol). Quantification based on an external calibration using galacturonic acid.

Contents of methyl- and acetyl-esters were analyzed using the method described by Müller-Maatsch et al. (32). Sodium hydroxide (2 M) in deuterium oxide was added to IDF and SDF for ultrasonic-assisted saponification (2 h). After filtration (PTFE, 0.45 μm) supernatants were analyzed by ^1^H-nuclear magnetic resonance (NMR) spectroscopy using an Ascend 500 MHz NMR spectrometer (Bruker, Rheinstetten, Germany) equipped with a Prodigy cryoprobe. Measurements were performed using the zg30 pulse sequence and a relaxation delay (D1) of 35 s with 32 scans. For referencing of ^1^H-NMR spectra and as internal standard for the quantification of released methanol (δ_H_ = 3.36 ppm) and acetic acid (δ_H_ = 1.92 ppm), 3-(trimethylsilyl)propionate-*d4* (δ_H_ = 0.00 ppm) was used.

#### 2.3.8 Molecular weight distribution of SDF

Molecular weight distribution was analyzed by HPSEC-RID as previously described by Schmid et al. (33). In brief, SDF of untreated and treated pea hulls were dissolved in 50 mM sodium nitrate (2 g/L) for 24 h at 37 °C. After centrifugation, oligo- and polysaccharides of the supernatant (50 μL) were separated based on their hydrodynamic volume on a guard column (TosohTSK-gel PW_XL_ 40 mm × 6.0 mm, 12 μm particle size, Tosoh) and two size-exclusion columns in series (TosohTSKgel G6000PW_XL_ 300 mm × 7.8 mm, 13 μm particle size, TSKgel G4000PW_XL_ 300 mm × 7.8 mm, 10 μm particle size, Tosoh) by isocratic elution with sodium nitrate (50 mM) at 50 °C with a flow rate of 0.5 mL/min. Dextran standards (5–670 kDa) were used for calibration.

#### 2.3.9 Analysis of oligosaccharides in the LMWSDF-fraction

Oligosaccharides are the main components of the LMWSDF-fractions, but depend on their solubility in 78% ethanol. The arabino-, cello-, galacturonic acid-, and xylooligosaccharides were analyzed by HPAEC-PAD(-MS) on an analytical CarboPac PA200 column at 25 °C with a flow rate of 0.4 mL/min. Ultra-pure water (A), 100 mM sodium hydroxide (B), and 100 mM sodium hydroxide with 500 mM sodium acetate were used as eluents. Before injection of the diluted LMWSDF-fractions (25 μL), the column was rinsed with 100% B (10 min) and equilibrated with 90% A and 10% B (20 min). Separation was performed using the following gradient: 0–20 min linearly to 35% A and 65% B; 2–28 min linearly to 35% A, 63.5% B, and 1.5% C; 28–32 min isocratic conditions; 32–55 min linearly to 35% A, 55% B, and 10% C; 55–57 min linearly to 16% A, 60% B, and 24% C; 57–67 min linearly to 70% B and 30% C; 67–72 min linearly to 45% B and 55% C; 72–82 min linearly to 40% B and 60% C; and finally rinsing the column with 100% C for 15 min. External calibrations for each oligosaccharide and isomaltotriose as internal standard (final concentration: 10 mM) were used for quantification. For identification of oligosaccharides according to their mass-to-charge ratios (*m/z*) selected samples were analyzed with HPAEC-PAD-MS based on the principle described by Steck et al. (30). In brief, the eluate was desalted with an electrolytic regenerating suppressor (Dionex AERS 500 and AXP pump, Thermo Fisher Scientific) in reverse flow with ultrapure water, and lithium chloride (0.5 mM) was added to form lithium adduct-ions in the following electron spray ionization (ESI).

For a detailed characterization of the released galacturonic acid oligosaccharides, the LMWSDF-fractions were additionally analyzed with hydrophilic interaction liquid chromatography (HILIC) using an ultra-high performance liquid chromatography (UHPLC)-MS system (Nexera X2, LCMS 2020, Shimadzu). Chromatography was performed on an amide-based stationary phase (Acquity UPLC BEH Amide, 150 x 2.1 mm, 1.7 μm particle size, Waters Corporation, Eschborn, Germany) using a modification of the method published by Leijdekkers et al. (34). Separation of diluted LMWSDF-fractions (10 μL) was achieved at 50 °C with a flow rate of 0.4 mL/min and the following gradient using acetonitrile:water (20:80, A) and acetonitrile:water (80:20, B) each with 10 mM ammonium formiate and 0.2% formic acid as eluents: 0.1–1 min with 100% B; 1–31 min linear gradient to 20% B; 31–35 min isocratic conditions; 35–36 min linear adjustment back to 100% B; and flushing the column with 100% B for 5 min. ESI was used in the negative mode, and MS was performed in the single ion monitoring (SIM)-mode for specific detection of galacturonic acid oligosaccharides (DP ≤ 10) with up to two methyl esters.

#### 2.3.10 Particle size distribution

The particle size distributions (PSD) of the untreated and treated pea hulls were measured by laser diffraction using an LA-950 analyzer (Horiba, Retsch Technology GmbH, Haan, Germany). The 50^th^ and 90^th^ percentiles (D_50_ and D_90_) were calculated from the volume-based PSD by Fraunhofer optical model using the instrument's software.

#### 2.3.11 Sedimentation

To determine the sedimentation of the untreated and mechanically-enzymatically treated pea hull suspensions, aliquots (10 mL) were transferred to test tubes and left undisturbed to allow for particle settling. The volume of the upper clear phase (V, mL) was measured several times (1 min to 20 h) and sedimentation percentage was calculated by $\frac{V}{10}*$100.

#### 2.3.12 Viscosity

The viscosity of mechanically(-enzymatically) treated pea hull suspensions was measured at 20 °C within 24 h after mechanical(-enzymatic) treatment under shear rate-controlled conditions using a rheometer (MCR 502, Anton Paar GmbH, Ostfildern, Germany) equipped with a C-PTD200 measuring cup (diameter: 28.9 mm) and a CC27/P6 36734 coaxial cylinder (diameter: 26.7 mm). Shear rates ranged from 1 to 1,000 s^−1^, and data points were recorded every 20 s. Only those suspensions that remained physically stable, i.e., without visible sedimentation for at least 10 min (duration of the measurement), were included in the analysis. Prior to viscosity analysis, suspensions were stirred for at least 10 min at 300 rpm.

#### 2.3.13 Water retention capacity

Water retention capacity (WRC) of mechanically-enzymatically treated pea hull suspensions was determined by a modification of the method described by Robertson et al. (35). In brief, aliquots (25 g) of fresh untreated and treated pea hull suspensions were centrifuged (3,000 g, 20 min). The supernatants were carefully decanted, and excess of liquid was removed by turning the tubes up-side down on a fine-meshed paper for 10 min. Sample weights were recorded immediately (m_hydrated_) and after oven-drying overnight at 105 °C (m_dried_). The WRC (g water/g insoluble mass) was calculated as the mass of water retained (m_hydrated_ - m_dried_) in reference to insoluble mass, which represents the mass of all water insoluble compounds.

## 3 Results and discussion

### 3.1 Characterization of untreated pea hull

The pea hull contained 76% DF, consisting mainly of IDF (70%) and lower amounts of SDF (5%) and LMWSDF (< 1%). The pea hull also contained protein (7%) and ash (3%). The RFO stachyose (0.3%), verbascose (0.3%), and raffinose (0.2%), contributing to LMWSDF, as well as the digestible carbohydrates glucose (0.1%), fructose (0.1%), sucrose, maltose, maltotriose, and starch were detected and partially quantified. Thus, the contents of total DF and IDF were higher than the ones described by Saldanha do Carmo et al. (19).

**Figure 2:**
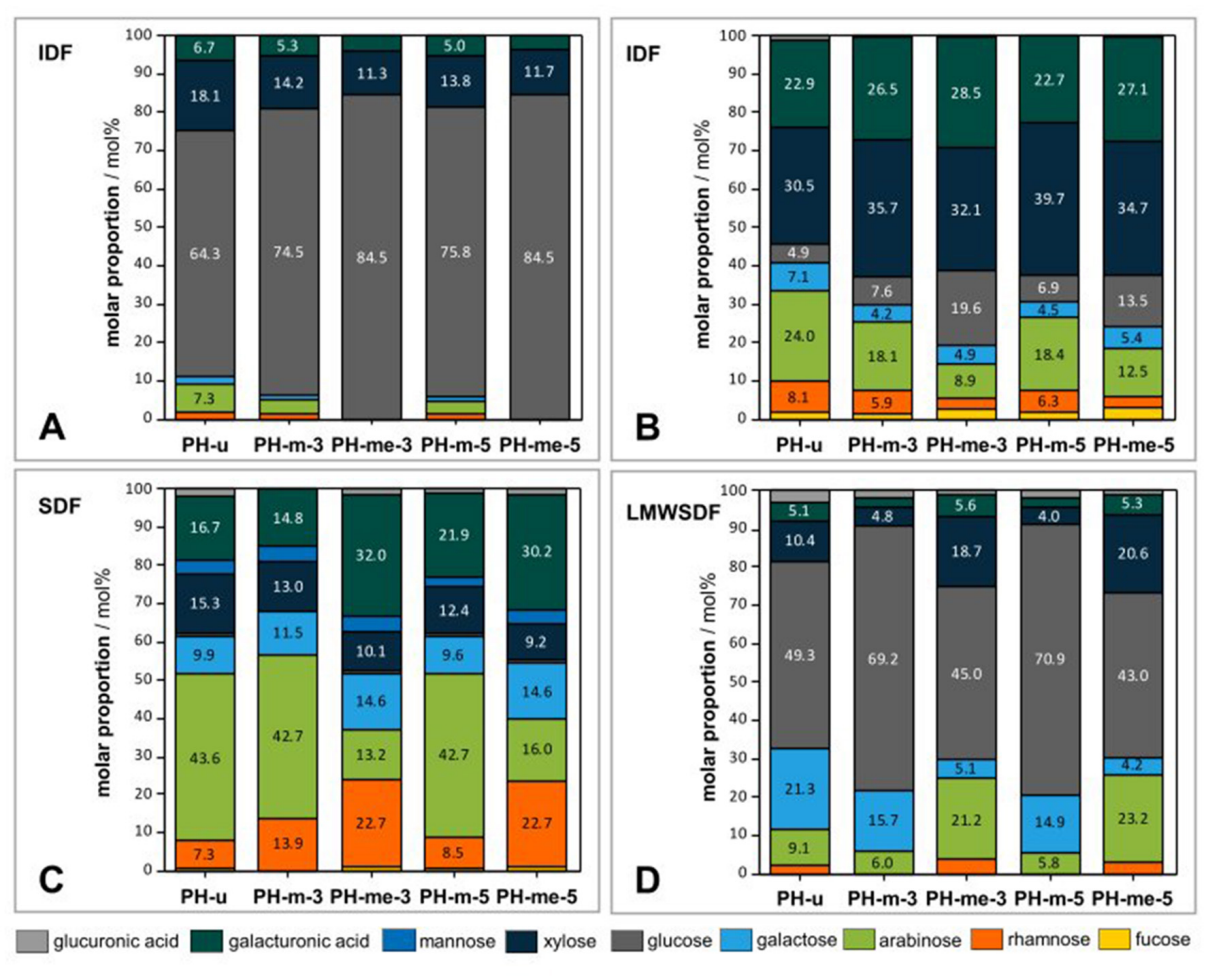
Monosaccharide composition (mol%) after (A) sulfuric acid hydrolysis of insoluble dietary fiber (IDF), and after methanolysis and trifluoroacetic acid hydrolysis of (B) IDF, (C) soluble (SDF), and (D) low-molecular weight soluble dietary fiber (LMWSDF)-fractions of untreated (PH-u), mechanically (PH-m-3/PH-m-5), and mechanically-enzymatically treated pea hulls (PH-me-3/PH-me-5) (*n* = 2; analytical replicates).

The monosaccharide compositions of pea hull IDF and SDF are shown in Figures 2A–C (and Supplementary Table S5), suggesting different polysaccharide profiles in these fiber fractions. The monomer composition of IDF polysaccharides after hydrolysis with sulfuric acid was comparable to the literature (36). Glucose dominated (64 mol%) after sulfuric acid hydrolysis, while only 5 mol% glucose was released from IDF polysaccharides by methanolysis and TFA-hydrolysis. It is therefore assumed that most of the glucose released by sulfuric acid originates from crystalline cellulose, which is hardly accessible for methanolysis and TFA-hydrolysis (25, 37). Methylation analysis data (Table 1) also showed that the PMAA of glucose (50 mol%) dominated in the IDF polysaccharides: 89% of the glucose originated from 1,4 glycosidic linkages and 1% from terminal ends, both occurring in cellulose. However, 1,4-linked and terminal glucose can also originate (partially) from xyloglucans. Apart from glucose, xyloglucans are also composed of xylose, galactose, and fucose, which were detected after methanolysis and TFA-hydrolysis of IDF. Also, methylation analysis revealed typical xyloglucan constituents (terminal, 1,4- and 1,4,6-linked glucose; terminal and 1,2-linked xylose; terminal galactose). Although fucose was not detected in the methylation analysis, results of the xyloglucan profiling approach (Supplementary Table S6) indicate that the majority of the enzymatically released xyloglucan oligosaccharides were fucosylated (48 mol%), with 46 mol% of the released oligosaccharides being only substituted with xylose. A small portion of the enzymatically released xyloglucanoligosaccharides (6 mol%) represented galactose containing xyloglucan segments. Based on these results the degree of substitution of the xyloglucans was calculated to be 73%, which is typical for dicotyledons (38). Overall, however, the xyloglucan content in the IDF was estimated to be low, based on the low proportion of characteristic PMAA (1,4,6-linked glucopyranose, 1,2-linked xylopyranose). Glucose is only of minor significance in the SDF monomer composition (Figure 2A) and only very small amounts of xyloglucans are assumed based on the results of the monosaccharide analysis, methylation analysis, and xyloglucan profiling (data not shown).

**Table 1:** Molar distribution of partially methylated alditol acetates (PMAA) obtained from polysaccharides of insoluble (IDF) and soluble dietary fiber (SDF) of pea hull by methylation analysis in mol% ± range/2 (*n* = 2, analytical replicates).

PMAA	Molar distribution/mol%	
	IDF	SDF
t-Glc*p*	0.5 ± 0.1	0.8 ± 0.0
1,4-Glc*p*	44.8 ± 0.0	1.5 ± 0.0
1,4,6-Glc*p*	2.5 ± 0.1	-
1,2,3,4-Glcp^*^	0.3 ± 0.0	-
1,2,3,4,6-Glcp^*^	2.1 ± 0.1	-
**sum**	**50.2** **±0.2**	**2.3** **±0.0**
t-Ara*f*	1.9 ± 0.0	6.5 ± 0.0
1,2-Ara*f*	0.2 ± 0.0	0.9 ± 0.0
1,5-Ara*f*/1,4-Ara*p*	7.8 ± 0.3	50.0 ± 0.5
1,3,5-Ara*f*	0.4 ± 0.1	0.8 ± 0.0
1,2,5-Ara*f*	1.1 ± 0.0	3.3 ± 0.0
1,2,3,5-Ara*f*	0.7 ± 0.1	0.7 ± 0.0
**sum**	**12.1** **±0.4**	**62.1** **±0.4**
t-Gal*p*	1.3 ± 0.0	3.5 ± 0.1
1,2-Gal*p*	-	0.8 ± 0.0
1,3-Gal*p*	-	1.2 ± 0.0
1,4-Gal*p*	0.4 ± 0.1	0.8 ± 0.4
1,6-Gal*p*	-	1.3 ± 0.1
1,3,6-Gal*p*	-	1.9 ± 0.0
**sum**	**1.7** **±0.1**	**9.6** **±0.4**
1,2-Rha*p*	0.9 ± 0.0	3.2 ± 0.0
1,2,4-Rha*p*	0.5 ± 0.0	1.8 ± 0.0
**sum**	**1.4** **±0.1**	**5.0** **±0.0**
t-Xyl*p*	2.7 ± 0.1	3.3 ± 0.0
1,2-Xyl*p*	0.4 ± 0.1	-
1,4-Xyl*p*	27.3 ± 0.8	15.4 ± 0.0
1,2,4-Xyl*p*	0.8 ± 0.0	0.8 ± 0.0
1,2,3,4-Xylp^*^	3.1 ± 0.1	-
**sum**	**34.2** **±0.7**	**19.5** **±0.04**
t-Man*p*	-	1.2 ± 0.0
1,3-Man*p*	-	0.3 ± 0.1
1,4-Man*p*	0.5 ± 0.0	-
**sum**	**0.5** **±0.0**	**1.5** **±0.0**

Ara, arabinose; Glc, glucose; Gal, galactose; Man, mannose; Rha, rhamnose; Xyl, xylose; f, furanose; p, pyranose; t, terminal. Bold values indicate the sum of glucose, galactose, rhamnose, xylose, or mannose derived PMMA, respectively.

^*^Presumably result of undermethylation.

The most abundant monosaccharide released after methanolysis and TFA-hydrolysis of IDF was xylose (31 mol%). A similar proportion was determined for PMAA of xylose (34 mol%) after methylation analysis. Since pea hulls contain many cells with a secondary cell wall, it was assumed that the detected xylose originated mainly from (glucurono-)xylans. In agreement, the dominant linkage type of xylose was the 1,4-linkage (Table 1), as present in linear xylans. The PMAA of 1,2,4-linked xylopyranose (0.8 mol%) indicated a few segments of substituted xylans. A substitution with glucuronic acid, which was released in traces from the polysaccharides of IDF by methanolysis and TFA-hydrolysis is likely. Based on the monosaccharide composition and methylation analysis data, the presence of (glucurono-)xylans is also assumed for SDF. Consistent with these results, the occurrence of linear xylans and low-substituted (arabinoglucurono-)xylans in water-soluble polysaccharides from pea hull was previously described (39).

Homo- and type I rhamnogalacturonan monomers (arabinose, galactose, galacturonic acid, and rhamnose) were released from IDF and SDF polysaccharides (Figures 2A–C, Table 1). The ratio of homogalacturonans to type I rhamnogalacturonans, calculated from the molar proportions of galacturonic acid and rhamnose after Broxterman et al. (40), was balanced (ratio: 0.9) in IDF, whereas type I rhamnogalacturonans are dominant in SDF (ratio: 0.6). Specific evidence for the occurrence of xylogalacturonans, as described by Renard et al. (41) and (36)), was not provided here. The galacturonic acid units in homogalacturonans, type I rhamnogalacturonans, and xylogalacturonans can be methylated and/or acetylated. The low DM (IDF: 0.1%, SDF: 0.2%) and DAc (IDF/SDF: 0.1%) determined here, indicated, however, very few ester bonds in the present pectin structures. In contrast, 36) described higher DM (33%) and DAc (40%) for polymer-bound galacturonic acids in pea hulls. About two-thirds of the rhamnose units of the type I rhamnogalacturonan backbones were unsubstituted (1,2-linked) in both IDF and SDF. Consequently, about one-third of the rhamnose backbone units were substituted (1,2,4-linked) with either arabinans and/or (arabino-)galactans.

The neutral side-chains of type I rhamnogalacturonans in IDF and SDF were dominated by arabinans, as indicated by the higher molar proportions of arabinose after acid hydrolysis in comparison to galactose, being in accordance with literature (39). In SDF, arabinans even dominated the overall polysaccharides composition (44 mol% arabinose). The structures of the insoluble and soluble arabinans were comparable according to the results of the methylation analysis (Table 1) and the arabinan profiling (Table 2). In the following, the arabinan structures will be explained more detailed, taking the insoluble arabinans as an example. They were mainly linear, which was represented by the PMAA of the 1,5-linked arabinofuranose (8 mol%) and the enzymatically released α-(1 → 5)-linked arabinobiose (A2a, 85 mol%) in the arabinan profiling. However, if side-chains occur, they are mainly located in position *O*2- ( ≤ 1 mol% 1,2,5-linked arabinofuranose/12 mol% arabinotetraose A4b) and to a lesser extend in position *O*3- ( ≤ 1 mol% 1,3,5-linked arabinofuranose/1 mol% arabinotetraose A4a and 2 mol% arabinoheptaose A7b) as well as in *O*2- and *O*3-position ( ≤ 1 mol% 1,2,3,5-linked arabinofuranose/traces of arabinopentaose A5a). The observed linear arabinan structure was also found in water-soluble polysaccharides of pea hull by Ralet et al. (39). In contrast, however, these authors detected more side-chains in the *O*3- than in *O*2-position and also fewer double-substituted arabinofuranose units.

**Table 2:** Molar distribution (mol%) of enzymatically released arabino- and galactooligosaccharides of insoluble (IDF) and soluble dietary fiber (SDF) from pea hull during arabinan and galactan profiling ± range/2 (*n* = 2, analytical replicates); A2a, A4a, … and G2a, nomenclature of arabino- and galactooligosaccharides according to Wefers and Bunzel (16).

	Molar distribution/mol%	
	IDF	SDF
Arabinooligosaccharides		
A2a	84.6 ± 0.0	84.9 ± 0.3
A4a	1.1 ± 0.0	1.7 ± 0.0
A4b	12.0 ± 0.0	9.7 ± 0.2
A5a	< LOQ	0.5 ± 0.0
A6a	-	1.5 ± 0.0
A7b	2.3 ± 0.0	1.7 ± 0.1
Galactooligosaccharides		
G2a	100.0 ± 0.0	-

LOQ, limit of quantification.

The (arabino-)galactans in the IDF were mainly linear and galactose based as shown by the detection of terminal and 1,4-linked PMAA of galactopyranose (methylation analysis) and β-(1 → 4)-linked galactobiose (G2a) [galactan profiling (Table 2)]. Methylation analysis of SDF released PMAA of galactose that are typical for (arabino-)galactans type I and II (terminal, 1,4-; 1,3-, 1,3,6-linked). The galactan profiling approach, however, did not confirm the presence of (arabino-)galactans type I. While this conflicting information cannot be clearly resolved, there are surely other galactose sources in the SDF contributing to the galactose content as measured after acid hydrolysis (Figure 2C), e.g., RFO, (arabino-)galactans type II, and xyloglucans. In fact, the presence of raffinose, stachyose, and verbascose was verified by analysis with HPAEC-PAD.

Overall, pea hull fiber polysaccharides primarily consisted of cellulose followed by (glucurono-)xylans, low-esterified pectins, and xyloglucans. Major pectic elements are homogalacturonans and type I rhamnogalacturonans, especially mainly linear arabinans. Consequently, the focus of the enzymatic hydrolysis of the pea hull was set on the degradation of cellulose, (glucurono-)xylans, arabinans, and homogalacturonans. For this purpose, appropriate enzymes (cellulase, xylanase, arabinanase, polygalacturonanase) were tested and characterized.

### 3.2 Characterization of enzymes

The results of the characterization (optimum temperature, side activities, conditions of inactivation) of commercial cellulase, xylanase, arabinanase, and polygalacturonanase, which could be suitable for enzymatic hydrolysis of pea hull, are listed in Table 3. The optimum temperature of the four enzymes was around 40–50 °C. However, arabinanase also performed well at lower temperature (30 °C) and xylanase at higher temperature (60 °C). With cellulose being the major target of the hydrolysis, the enzymatic hydrolysis was carried out at 50 °C. When characterizing the side-activities of the enzymes, only the polysaccharides that were present in relevant amounts in the pea hull [cellulose, (glucurono-)xylans, arabinans, and homogalacturonans] were considered. Except *endo*-polygalacturonanase, all characterized enzymes showed diverse side-activities of varying strength. Most importantly, cellulase also showed *exo*-polygalacturonanase- and xylanase-activities, and xylanase showed cellulase-activity. Therefore, when optimizing enzymatic hydrolysis, we focused on the mass of commercial enzyme preparations rather than on individual enzyme activities, with the aim of achieving high levels of LMWSDF and oligosaccharides in each case. A 10-min heat treatment at 95 °C was sufficient to eliminate most enzyme activities (Table 3) of commercial cellulase, arabinanase, and polygalacturonanase which were accordingly applied in the experimental design for the mechanical-enzymatic treatment.

**Table 3:** Characteristics (optimum temperature, side-activities, conditions of inactivation) determined for the commercial enzymes used for enzymatic hydrolysis of pea hull.

Enzymes	Optimum temperature	Side-activities	Conditions of inactivation
**Arabinanase**	30–40 °C	xylanase	80 °C (10 min) + 1,750 bar (5 runs) ** → active** 95 °C (10 min) ** → inactive**
**Cellulase**	50 °C	arabinanase, *exo*-polygalacturonanase, xylanase	80 °C (10 min) + 1,750 bar (5 runs) ** → active side activities** 95 °C (10 min) ** → inactive**
***Endo*** **-poly-galacturonanase**	40 °C	-	80/95 °C (10 min) ** → inactive**
**Xylanase**	50–60 °C	cellulase	80 °C (10 min) + 1,750 bar (5 runs)/95 °C (10 min) ** → active** 80% ethanol ** → reversible inactive**

### 3.3 Optimization of the mechanical-enzymatic treatment

The aim of the mechanical-enzymatic treatment of the pea hull was to obtain physically stable oligosaccharide-rich fiber preparations. Also, application of different enzyme mixtures was supposed to influence the oligosaccharide profile. Initial high-pressure treatment was intended to mechanically loosen the cell wall network and increase the surface area by reducing the particle size. Both were intended to improve subsequent enzymatic hydrolysis due to the improved accessibility of the polysaccharides. Microfluidization using one, three, or five runs (PH-m-1/3/5) did not result in major chemical modifications of the fiber polysaccharides (compared to the untreated pea hull PH-u), as concluded from the performed analyses (DF analysis, monomer composition of the polysaccharides, molecular weight distribution, and arabinan composition (Supplementary Tables S7–S9 and Supplementary Figure S1). But, mechanical treatment improved the physical stability of the pea hull suspensions.

Testing the different enzyme preparations on mechanically treated pea hulls (PH-m-1/3/5) showed that the addition of cellulase was found to be necessary to improve the activity of the other enzymes as could be concluded from the molar distribution of the poly- and oligosaccharides following different enzymatic treatments (Supplementary Figure S2A) and, for example, an improved production of degradation products of arabinans by the combined use of arabinans and cellulase. Presumably, enzymatic degradation of cellulose improved accessibility of other enzymes to the polysaccharides. Differently, monitoring different approaches of enzymatic hydrolysis showed that the addition of polygalacturonanase only slightly increased the degradation of pea hull fiber polysaccharides as indicated by the molar distribution of the poly- and oligosaccharides of the corresponding mechanically-enzymatically treated pea hulls (Supplementary Figure S2B) and no detectable oligogalacturonic acids. Hence, polygalacturonanase was no longer considered in the mechanical-enzymatic approach. Thus, the monitoring results suggested to focus on cellulase and arabinanase to degrade pea hull fiber polysaccharides (Supplementary Figure S2C); xylanase was omitted as it was hard to inactivate (Table 3). Additionally, the monitoring showed promising results using a hydrolysis time of max. 30 min. Considering again the molar distribution, the degradation of the polysaccharides was more advanced after 30 and 60 min compared to 10 min (Supplementary Figure S2D), the degradation was only slightly improved after 60 min compared to 30 min. But, a longer hydrolysis time (60 min) led to an increased ratio of mono- and disaccharides to oligosaccharides compared to shorter hydrolysis times (10/30 min). As it was assumed that a more intensive mechanical treatment of the pea hull results in improved subsequent enzymatic hydrolysis (Supplementary Figure S2E), the two more intensively treated pea hulls (PH-m-3 and PH-m-5) were hydrolyzed for 30 min with cellulase and arabinanase under the same conditions.

### 3.4 Characterization of mechanically-enzymatically treated pea hull

#### 3.4.1 Modifications in the fiber composition

**Figure 3:**
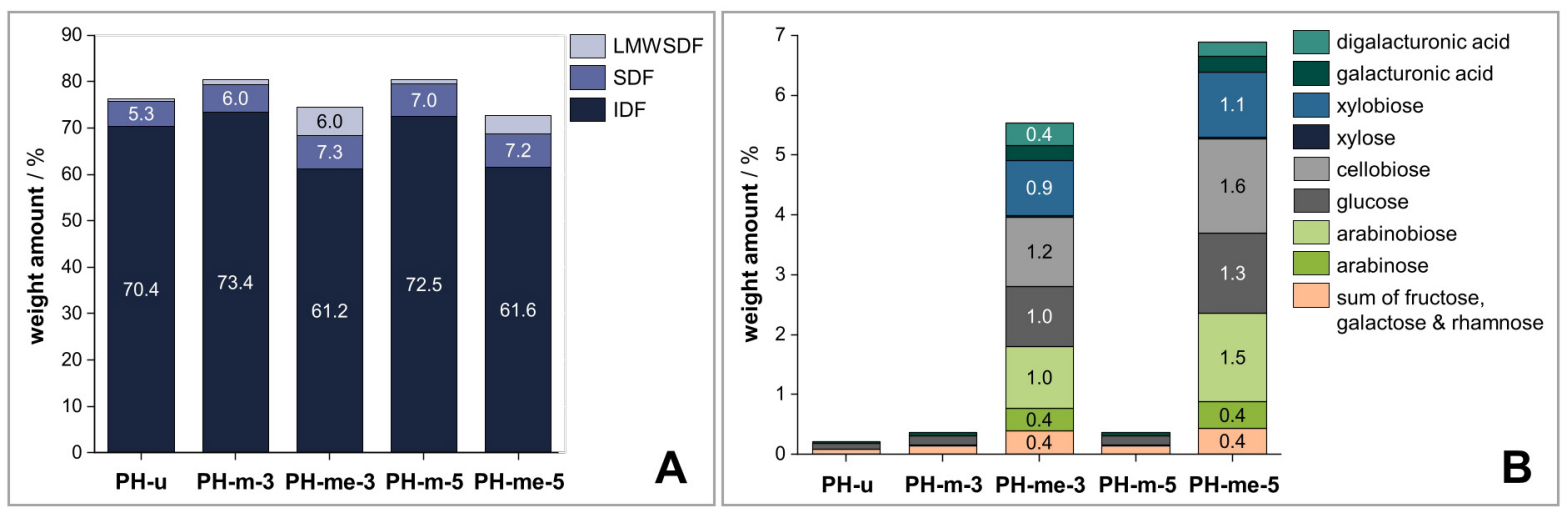
Weight amount (% of dried pea hull) of (A) dietary fiber separately for insoluble (IDF), soluble (SDF), and low-molecular weight soluble dietary fiber (LMWSDF) and of (B) free mono- and disaccharides of the untreated (PH-u), mechanically (PH-m-3/PH-m-5), and mechanically-enzymatically treated pea hulls (PH-me-3/PH-me-5) (*n* = 2; analytical replicates).

**Figure 4:**
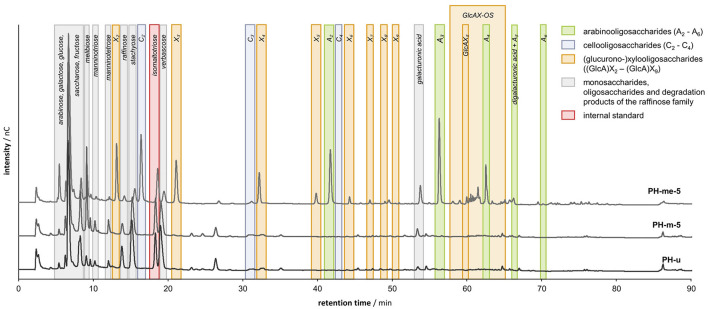
Chromatograms recorded by high-performance anion exchange chromatography with pulsed amperometric detection of the low molecular weight soluble dietary fiber fractions of untreated (PH-u), mechanically (PH-m-5), and mechanically-enzymatically treated pea hulls (PH-me-5); 2–9 = degree of polymerization.

**Figure 5:**
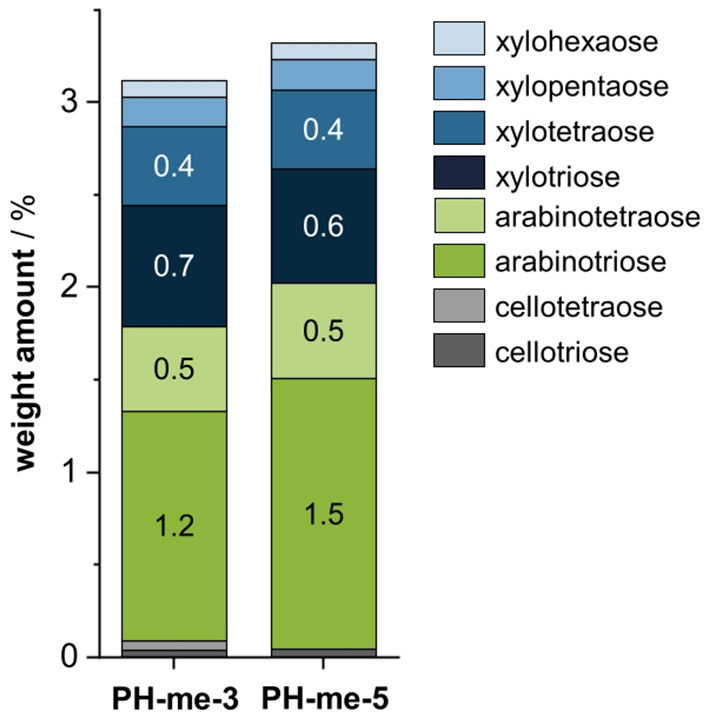
Weight amount (% of dried pea hull) of quantifiable oligosaccharides in the low-molecular weight soluble dietary fiber-fractions of the mechanically-enzymatically treated pea hulls (PH-me-3/PH-me-5) (*n* = 2; analytical replicates).

In contrast to the purely mechanical treatment, the combined mechanical-enzymatic treatments of the pea hull resulted in a slight reduction of total DF contents (Figure 3A and Supplementary Table S10). Especially the amount of IDF was reduced from 70% (PH-u) or 73% (PH-m-3 and PH-m-5), respectively, to about 61 % (PH-me-3 and PH-me-5). As a result, the amount of LMWSDF (from < 1% to 4%−6%) increased. However, we assume that the LMWSDF contents were even underestimated. As previously described (42), arabino- and xylotriose, which represented over 60% of the quantifiable oligosaccharides in the LMWSDF-fractions of PH-me-3 and PH-me-5 as determined by HPAEC-PAD (Figures 4, 5), are not or only partially detected as LMWSDF by the used AOAC method. At the same time, however, melibiose (a degradation product of raffinose, see below) is falsely recorded as LMWSDF. Varying degrees of decreases (18%−51%) in the amount of IDF and increases in alcohol-insoluble (3-4-fold, equal to SDF) and soluble mass (2-12-fold, containing LMWSDF, di-, and monosaccharides) are described in the literature (15) if the reverse order of treatments (first enzymatic treatment followed by a mechanical treatment) was applied to pea hulls. The stronger decrease in IDF reported by Morales-Medina et al. (15) may be related to the reversed treatment sequence (i.e., enzymatic + mechanical treatments) and different hydrolysis conditions. Enzymatic pre-treatment may first weaken accessible regions of the pea hull cell wall, thereby facilitating subsequent mechanical disruption and solubilization during high-pressure homogenization. In the present study, by contrast, microfluidization mainly reduced particle size, whereas the following enzymatic hydrolysis was probably restricted to more accessible surface regions of the cellulose-rich insoluble matrix. However, differences in enzyme composition and treatment conditions between both studies should also be considered, and the effect cannot be attributed exclusively to treatment order.

Besides changes in the individual fiber fractions, both mechanical-enzymatic treatments (PH-me-3 and PH-me-5) resulted in the release of mono- (approx. 2%) and disaccharides (approx. 4%), explaining the reduced total DF contents after the treatments. As shown in Figure 3B (and Supplementary Table S10), cellobiose, glucose, xylobiose, and arabinobiose dominate the released mono- and disaccharides, followed by arabinose, mono- and digalacturonic acid, and xylose. Although they are not legally defined as dietary fiber components, it should be noted that arabino-, cello- and xylobiose have been postulated to have various nutritional beneficial properties comparable to those of DF (7, 43, 44).

**Figure 6:**
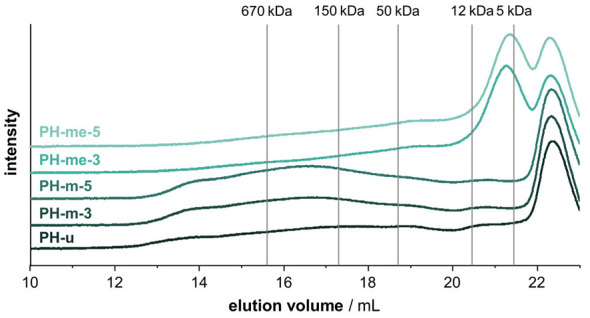
Molecular weight distribution of soluble dietary fiber of untreated (PH-u), mechanically (PH-m-3/PH-m-5), and mechanically-enzymatically treated pea hulls (PH-me-3/PH-me-5) as measured by using high performance size-exclusion chromatography with refractive index detection. Dextran standard substances (5–670 kDa) were used as molecular weight markers (*n* = 2; analytical replicates).

Mechanical and mechanical-enzymatic treatments of the pea hull resulted in modified molecular weight distributions of the SDF polysaccharides. SDF of PH-u mostly contained polysaccharides with a low molecular weight (< 5 kDa), while mechanical treatments led to a slight increase in soluble polysaccharides with a higher molecular weight (12 to >670 kDa, Figure 6), suggesting some solubilization or physical release of high molecular weight polysaccharides. Differently, a large increase of polysaccharides with a molecular weight below 12 kDa but larger than 5 kDa was monitored for the SDF of PH-me-3 and PH-me-5. At least partially, this might result from enzymatic hydrolysis of mechanically released high-molecular weight soluble polysaccharides. In agreement with our data, it was demonstrated for peach pomace that the average molecular weight of soluble polysaccharides decreased after mechanical-enzymatic treatment using a microfluidizer and commercial cellulase, resulting in a narrower molecular weight distribution (14).

#### 3.4.2 Modifications in the structure of polysaccharides

As a consequence of the mechanical-enzymatic treatments of the pea hull, cellulose, (glucurono-)xylans, arabinans, and homogalacturonans were enzymatically hydrolyzed. This was concluded based on the results of the analysis of free mono-, di- and oligosaccharides (Figure 3B and Figure 5) and of the monomer composition of IDF, SDF, and LMWSDF (Figure 2 and Supplementary Table S11) isolated from PH-me-3 and PH-me-5 in comparison to the results of PH-u, PH-m-3, and PH-m-5.

Glucose (1.6%; 1.2%), cellobiose (1.3%; 1.0%), and small amounts of cellooligosaccharides (DP = 3–4) were released from cellulose in PH-me-5 and PH-me-3, respectively. In IDF, however, cellulose remained predominantly in a crystalline form that was hardly hydrolysable (Supplementary Table S12).

Detection of (glucurono-)xylooligosaccharides (1.3%), xylobiose (approx. 1.0%), and xylose (traces) in PH-me-3 and PH-me-5 indicated the degradation of (glucurono-)xylans by the xylanase-activity of the cellulase. In accordance with this the molar proportions of xylose in the monomer composition of the LMWSDF-fractions were increased. Although some xylans were obviously hydrolyzed the monomer composition of IDF of PH-me-3 and PH-me-5 still contained larger amounts of xylose (Figures 2A, B, Supplementary Tables S11, S12). Based on these results the xylanase-side-activity of the cellulase was not sufficient to substantially degrade the (glucurono-)xylans. The incorporation of an additional xylanase that is easier to inactivate could improve xylan degradation. However, given the interactions between linear xylans and cellulose in secondary plant cell walls, these xylans are likely to be difficult to degrade completely by enzymatic means. Mechanical-enzymatic treatments of pea hulls resulted in a pronounced degradation of arabinans. This was concluded from the amounts of released arabinooligosaccharides (approx. 2.0%), arabinobiose (1.0%−1.5%), and arabinose (0.4%), which were slightly higher in PH-me-5 than in PH-me-3. Accordingly, the molar fraction of arabinose in the monomer composition of the LMWSDF-fractions was more than twice as high (21–23 mol%) after mechanical-enzymatic treatments. Also, the monomer compositions of IDF and SDF showed reduced amounts of arabinose after mechanical-enzymatic treatments compared to PH-u, PH-m-3, and PH-m-5 (Figures 2A–C). The arabinan profiling (Supplementary Table S13) indicated a linearization of the arabinan structures as the molar proportions of enzymatically released arabinobiose (A2a) increased, whereas those of the substituted arabinooligosaccharides (A4a, A4b, A5a, A7a, and A7b) decreased. Hence, it was assumed that the used enzymes contained an arabinofuranosidase-activity. However, mechanical treatment already led to an increased (relative) release of A2a, potentially by non-enzymatic, hydrolytic cleavage of substituted arabinose units from arabinans favored by slightly acidic pH (5.0–5.7), high-pressure, and higher temperature during the mechanical treatment. Comparing the degradation of arabinans during mechanical-enzymatic treatments in IDF and SDF based on the relative proportions of arabinose in the monomer compositions (Figures 2A–C), it appears that soluble arabinans are degraded more efficiently than insoluble arabinans. To a lesser extent the degradation of homogalacturonans by *exo*-polygalacturonanase-activity of cellulase was observed by the detection of small amounts of mono-, di- and oligogalacturonic acid.

Overall, degradation of the major polysaccharides of the pea hull was largely comparable between the two mechanical-enzymatic treatments. Thus, no markedly greater loosening of cell wall structures by a more intensive mechanical pre-treatment was observed.

#### 3.4.3 Analysis of oligosaccharides in the fractions of LMWSDF

The LMWSDF-fractions of PH-u, PH-m-3, and PH-m-5 contained only RFO (DP = 3–5) and their respective degradation products after release of fructose (DP = 2–4; melibiose, manninotriose, manninotetraose) as concluded from the analysis by HPAEC-PAD-(MS) (5). Melibiose, manninotriose, and manninotetraose were identified by HPAEC-PAD/MS using the *m/z* and retention times in comparison to a RFO standard mixture before and after enzymatic hydrolysis with invertase. An absolute quantification was not performed. However, the signal intensities of RFO and their hydrolysis products from LMWSDF-fractions of PH-me-3 and PH-me-5 were comparable to those of PH-m-3 and PH-m-5 indicating comparable amounts. But, the signal intensity of melibiose increased with increasing intensity of the mechanical treatment, while signal intensities of all RFO, manninotriose, and manninotetraose remained constant. Hence, a non-enzymatic, hydrolytic release of melibiose from raffinose favored by a lower pH (5.0–5.7), high-pressure, and elevated temperatures during mechanical(-enzymatic) treatment of the pea hull appears possible.

The oligosaccharide profiles of PH-me-3 and PH-me-5 were dominated by arabino- and xylooligosaccharides (Figure 5 and Supplementary Table S14). Arabinotriose and -tetraose made up about 60% and xylotriose to -hexaose about 40% of the quantifiable oligosaccharides. In addition, arabinopentaose and -hexaose as well as longer-chain xylooligosaccharides (DP = 7–12) and glucuronoxylooligosaccharides were detected by HPAEC-PAD(-MS) (5). In total, the release of short-chain oligosaccharides (DP = 3–4) was favored indicating a preferred enzymatic hydrolysis of previously released longer-chain oligosaccharides. However, the broad spectrum of DP of the (glucurono-)xylooligosaccharides indicated an incomplete degradation of (glucurono-)xylans, potentially due to the substitution with glucuronic acid. The proportions of cellotriose and -tetraose were negligible (< 0.1% each), and cellopentaose and -hexaose were only detected qualitatively. As larger amounts of cellobiose and glucose were found, these data demonstrate an efficient degradation of liberated higher oligosaccharides. Also, partially methyl-esterified galacturonic acid oligosaccharides (DP = 3–5, 9) from the degradation of homogalacturonans, which were detected by HILIC-MS, were of minor relevance (data not shown).

When comparing the two differently processed pea hulls, the oligosaccharide profiles of the LMWSDF-fractions of PH-me-3 and PH-me-5 were similar. This supports the assumption that the more intensive mechanical pre-treatment of the pea hull did not result in an improved enzymatic accessibility of the polysaccharides due to a more pronounced mechanical loosening of the cell wall structures and mechanical disruption of particles.

#### 3.4.4 Analysis of particle size distribution, sedimentation, viscosity, and water retention capacity

**Figure 7:**
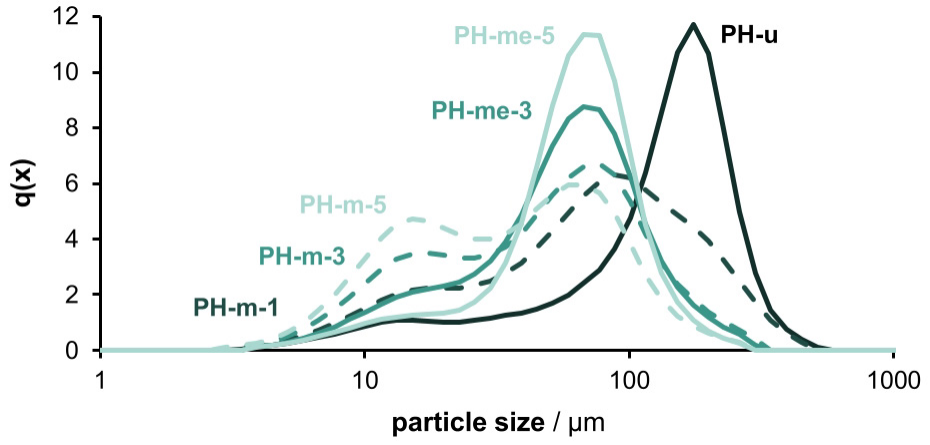
Volumetric based particle size distributions [q(x)] of untreated (PH-u), mechanically (PH-m-3/PH-m-5) and mechanically-enzymatically treated (PH-me-3/PH-me-5) pea hull suspensions as determined by laser diffraction (*n* = 6, two batches in analytical triplicates).

The volumetric PSD of the five analyzed suspensions are shown in Figure 7 and their respective D_50_ and D_90_ values are listed in Supplementary Table S15. PH-u exhibited a broad monomodal distribution (D_50_ = 142.2 μm; D_90_ = 242.7 μm). In contrast, PH-m-3 and PH-m-5 showed a bimodal distribution with peaks at approx. 20 and 80 μm and significantly reduced particle sizes, with D_50_ values of 46.9 μm and 33.9 μm, respectively. Pea hull particles are primarily composed of cell wall fragments rich in cellulose, organized as bundles of macrofibers, microfibers, and fibrils (45, 46). Microfluidization generates intense turbulence, exposing these structures to high shear and cavitation forces (47). Under these conditions, cellulose undergoes defibrillation, progressively breaking down from macrofibers into smaller fibrillar structures. Despite the substantial particle size reduction, the IDF content remained nearly unchanged (Figure 3A), consistent with previous reports indicating no significant decrease in insoluble mass (which is comparable to IDF) for microfluidized pea hull suspensions with D_90_ > 80 μm (13).

Combined mechanical-enzymatic treatments (PH-me-3 and PH-me-5) resulted in different largely monomodal PSD (Figure 7) with PH-me-5 showing a more pronounced monomodal distribution than PH-me-3. Both suspensions presented increased average D_50_ (57.3 μm) and D_90_ values (111.0 μm) compared to PH-m-3 and PH-m-5, respectively. This increase in particle size is attributed to the reduction of the number of particles with a size below 20 μm. The pronounced reduction of particles smaller than 20 μm suggests a preferential enzymatically catalyzed breakdown of smaller particles since enzymatic hydrolysis is facilitated by the larger surface area and lower steric hindrance compared to larger cell wall fragments such as cellulose macrofibers. During enzymatic hydrolysis, the total number of particles decreases, particularly those of smaller size, resulting in a particle size distribution shifted toward larger sizes. This effect was slightly more pronounced between PH-m-5 and PH-me-5 as compared to PH-m-3 and PH-me-3. A breakdown of smaller particles, likely accompanied by partial solubilization of cell wall polysaccharides, may have been reflected in the decrease of the IDF content from approx. 73% (PH-m-3 and PH-m-5) to approx. 61% (PH-me-3 and PH-me-5) after the enzymatic treatment (Figure 3).

As a result of the mechanical-enzymatic treatments, the technofunctional properties of the untreated and treated pea hull suspensions (i.e., WRC, sedimentation) were different (Table 4). The WRC refers to the capacity of insoluble mass to retain water (35). The WRC of dietary fiber is a key parameter for their application in food matrices (48). Water may be retained within fiber-rich particles either as free or bulk water, entrapped in porous structures, voids, or capillaries, or as bound water interacting with fiber surfaces via hydrogen bonding (49, 50). PH-u exhibited a low WRC (5.3 g water/g insoluble mass) due to the dense and compact structure of the cell wall, whereas PH-me-3 and PH-me-5 presented values of 15.0 and 25.2, respectively (Table 4). For solely microfluidized samples, the increase in WRC has previously been linked to particle size reduction and defibrillation of cellulose, which increase both the surface area and the number of exposed hydrophilic groups (13). In the present study, PH-me-3 and PH-me-5 showed similar particle size ranges, although PH-me-5 exhibited a somewhat lower D_90_ value and a more pronounced monomodal distribution (Figure 7 and Supplementary Table S15). Therefore, differences in particle size alone may not fully explain the higher WRC of PH-me-5. The increased water retention may also be associated with differences in the microstructure of the remaining insoluble matrix, potentially including variations in the extent of defibrillation, porosity, or exposure of hydrophilic sites after the more intensive mechanical treatment.

**Table 4:** Water retention capacity and sedimentation of untreated (PH-u), and mechanically-enzymatically treated pea hulls (PH-me-3/PH-me-5) ± standard deviation (*n* = 4, two batches in analytical duplicates).

	Water retention capacity /g water/g insoluble mass	Sedimentation/%	
		10 min	20 h
PH-u	5.3 ± 0.5	immediately	
PH-me-3	15.0 ± 4.8	immediately	
PH-me-5	25.2 ± 3.1	0.0	30.0 ± 2.8

**Figure 8:**
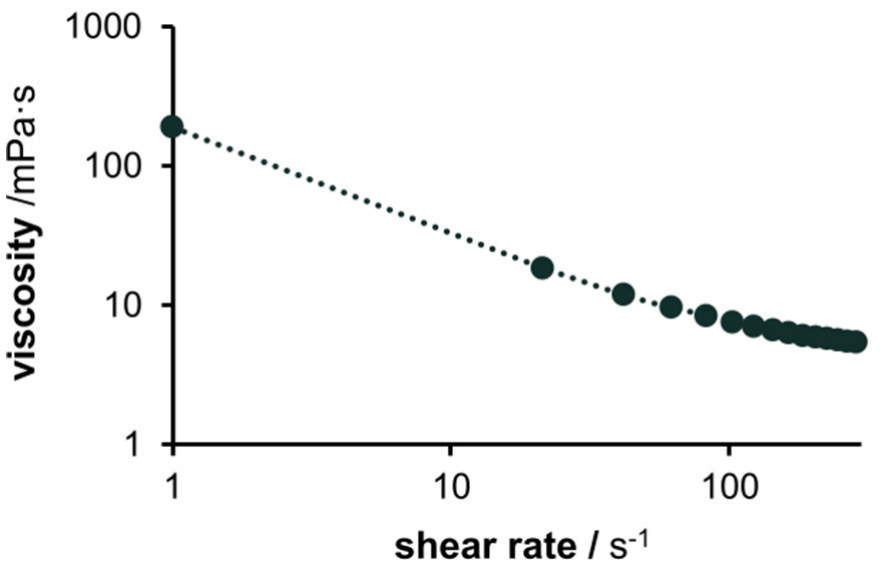
Viscosity as a function of shear rate of a 1 wt% mechanically-enzymatically treated pea hull suspension (PH-me-5) measured at 20°C (*n* = 4, two batches in analytical duplicates).

The differences in particle size and hydration also modified the sedimentation behavior and viscosity. PH-u and PH-me-3 sedimented rapidly whereas PH-me-5 demonstrated improved stability without observed sedimentation after 10 min and 30% sedimentation after 20 h (Table 4). Even though mechanically-enzymatically treated pea hull suspensions presented sedimentation, viscosity was determined for PH-me-5, since it was stable enough for analysis (Figure 8). It presented shear-thinning behavior, that is, the viscosity decreases with increasing shear rate. This behavior has been widely described for microfibrillated cellulose (51, 52), pectin solutions (53), and pea hull suspensions (13). Pea hull suspensions can be described as networks of cellulose-rich insoluble dietary fiber dispersed in an aqueous phase containing partial hydrolyzed pectin-rich soluble dietary fiber of variable molecular weight as well as oligo, mono-, and disaccharides. As a result, the viscosity of the suspensions depends on the behavior of both phases. As for the soluble compounds, the higher the molecular weight and the ramifications, the higher the viscosity (53). Consequently, since the enzymatic hydrolysis reduced the molecular weight of SDF (Figure 6), the contribution of the soluble compounds to viscosity is comparably minor. The microstructural features of the particles, their surface composition, size and concentration govern the strength and nature of interparticle interactions (54). Among these factors, concentration plays a crucial role, as the viscosity of microfibrillated cellulose suspensions follows either a power- or exponential-type proportion with the concentration (54). In this context, the enzymatic treatment reduced the IDF, a fact that also decreased the viscosity.

Particle size, viscosity, and sedimentation are interrelated properties, as changes in one property often influence the others. Accordingly, in pea hull suspensions, a reduction in the particle size has been associated with increased viscosity (13). Moreover, sedimentation behavior can be interpreted through Stokes' law, which describes the settling velocity of spherical particles in a Newtonian fluid as being directly proportional to the square of the particle diameter and inversely proportional to the fluid's viscosity. Despite the differences with the current suspensions (i.e., high concentration of particles, non-Newtonian fluid, interactions of the particles, and presence of soluble compounds), the results obtained for the suspensions are in agreement with that behavior. Although both mechanically-enzymatically treated pea hulls had a roughly comparable dietary fiber composition [mainly IDF; mainly cellulose followed by (glucurono-)xylans, pectins, xyloglucans] they differ in their functional properties, especially in their physical stability with PH-me-3 being less stable than PH-me-5. The lower physical stability observed for PH-me-3 compared to PH-me-5 was mainly attributed to differences in the particle size distribution (Figure 7 and Supplementary Table S15) and microstructural features.

In conclusion, it was demonstrated that the intensity of the mechanical pre-treatment did not clearly improve enzymatic hydrolysis of cellulose-rich, pea hull cell wall polysaccharides. Also, the mechanical treatment itself added little to the degradation of cell wall polysaccharides, but reduced particle size and affected the pea hull suspension functionality. Extensive enzymatic hydrolysis of the polysaccharides was limited by the high amount of hardly hydrolysable crystalline cellulose. Thus, it is assumed that the mechanical treatment did not drastically increase the amount of amorphous cellulose. Consequently, a conversion of IDF to SDF and/or LMWSDF by mechanical-enzymatic treatment was reached, but only to a limited extent. Nevertheless, the mechanically-enzymatically treated pea hulls consisted of about three quarters of dietary fiber and showed an increased WRC and modified sedimentation behavior. Corresponding suspensions had an improved sedimentation behavior and a shear-thinning behavior. Overall, the functional properties of the pea hull could be successfully improved by the mechanical-enzymatic treatment. However, incorporating the treated pea hulls into beverages is still difficult. But, the treated pea hulls appear to be suitable to enrich semi-solid food products like yogurts with dietary fiber. Also, solid food products such as dough-based and baked/thermally treated products (e.g., bread, cookies, and noodles), where enhanced hydration properties are desirable and only minor textural impact is required, can benefit from the application of treated pea hulls. But, incorporation of the treated fiber samples in actual food products still needs to be tested as the functional properties of the fiber samples may also depend on the composition of the different food products. Whether or not the additional efforts and costs of five cycles of mechanical treatment compared to three cycles are justified certainly depends on the specific application of the mechanically and enzymatically treated fibers; that is, to what extent an increased WRC or sedimentation stability is beneficial for the product. Future studies may investigate how alternative combinations of mechanical and enzymatic treatments influence particle microstructure and phase stability in order to further reduce viscosity and increase physical stability of pea hull suspensions.

## Data Availability

The original contributions presented in the study are included in the article/Supplementary material, further inquiries can be directed to the corresponding author.

## Glossary

DAc, degree of acetylation; DP, degree of polymerization; DM, degree of methylation; EFSA, European Food Safety Authority; ESI, electron spray ionization; FID, flame ionization detector; GC, gas chromatography; HILIC, hydrophilic interaction liquid chromatography; HPAEC, high performance anion exchange chromatography; HPSEC, high performance size-exclusion chromatography; IDF, insoluble dietary fiber; LMWSDF, low molecular weight soluble dietary fiber; MS, mass spectrometry; *m/z*, mass-to-charge ratio; PAD, pulsed amperometric detection; PMAA, partially methylated alditol acetates; PSD, particle size distribution; RFO, oligosaccharides of the raffinose family; RID, refractive index detector; SDF, soluble dietary fiber; SIM, single ion monitoring; TFA, trifluoroacetic acid; UHPLC, ultra-high performance liquid; chromatography; WRC, water retention capacity.

## References

[1] McCleary BV Sloane N Draga A Lazewska I. Measurement of total dietary fiber using AOAC method 2009.01 (AACC International Approved Method 32-45.01): evaluation and updates. Cereal Chem. (2013) 90:396–414. doi: 10.1094/CCHEM-10-12-0135-FI

[2] McDougall GJ Morrison IM Stewart D Hillman JR. Plant cell walls as dietary fibre: range, structure, processing and function. J Sci Food Agric. (1996) 70:133–50. doi: 10.1002/(SICI)1097-0010(199602)70:2<133::AID-JSFA495>3.0.CO;2-4

[3] Schweizer TF Würsch P. The physiological and nutritional importance of dietary fibre. Experientia. (1991) 47:181–6. doi: 10.1007/BF01945423 1848187

[4] Slavin J. Fiber and prebiotics: mechanisms and health benefits. Nutrients. (2013) 5:1417–35. doi: 10.3390/nu5041417 23609775 PMC3705355

[5] Holscher HD. Dietary fiber and prebiotics and the gastrointestinal microbiota. Gut Microbes. (2017) 8:172–84. doi: 10.1080/19490976.2017.1290756 28165863 PMC5390821

[6] Olano-Martin E Gibson GR Rastall RA. Comparison of the in vitro bifidogenic properties of pectins and pectic-oligosaccharides. J Appl Microbiol. (2002) 93:505–11. doi: 10.1046/j.1365-2672.2002.01719.x 12174051

[7] Prandi B Baldassarre S Babbar N Bancalari E Vandezande P Hermans D . Pectin oligosaccharides from sugar beet pulp: molecular characterization and potential prebiotic activity. Food Funct. (2018) 9:1557–69. doi: 10.1039/C7FO01182B 29437169

[8] EFSA. European Food Safety Authority. EFSA panel on dietetic products, nutrition, allergies. Scientific opinion on dietary reference values for carbohydrates and dietary fibre. EFSA J. (2010) 8:1462. doi: 10.2903/j.efsa.2010.1462

[9] Institute of Medicine. Dietary Reference Intakes for Calcium and Vitamin D. Washington, DC: The National Academies Press (2011). 21796828

[10] Ozturk OK Turasan H. Latest developments in the applications of microfluidization to modify the structure of macromolecules leading to improved physicochemical and functional properties. Crit Rev Food Sci Nutr. (2022) 62:4481–503. doi: 10.1080/10408398.2021.1875981 33492179

[11] Chen J Gao D Yang L Gao Y. Effect of microfluidization process on the functional properties of insoluble dietary fiber. Food Res Int. (2013) 54:1821–7. doi: 10.1016/j.foodres.2013.09.025

[12] He X Dai T Sun J Liang R Liu W Chen M . Disintegrating the structure and improving the functionalities of pea fiber by industry-scale microfluidizer system. Foods. (2022) 11:418. doi: 10.3390/foods11030418 35159568 PMC8834372

[13] Morales-Medina R Dong D Schalow S Drusch S. Impact of microfluidization on the microstructure and functional properties of pea hull fibre. Food Hydrocoll. (2020) 103:105660. doi: 10.1016/j.foodhyd.2020.105660

[14] Xu H Jiao Q Yuan F Gao Y. *In vitro* binding capacities and physicochemical properties of soluble fiber prepared by microfluidization pretreatment and cellulase hydrolysis of peach pomace. LWT - Food Sci Technol. (2015) 63:677–84. doi: 10.1016/j.lwt.2015.03.033

[15] Morales-Medina R Manthei A Drusch S. Enzymatic pre-treatment defines the water-binding and rheological properties of dynamic ultra-high-pressure homogenised pea hull suspensions. Food Hydrocoll. (2024) 157:110454. doi: 10.1016/j.foodhyd.2024.110454

[16] Wefers D Bunzel M. Arabinan and galactan oligosaccharide profiling by high-performance anion-exchange chromatography with pulsed amperometric detection (HPAEC-PAD). J Agric Food Chem. (2016) 64:4656–64. doi: 10.1021/acs.jafc.6b01121 27167141

[17] Urbat F Müller P Hildebrand A Wefers D Bunzel M. Comparison and optimization of different protein nitrogen quantitation and residual protein characterization methods in dietary fiber preparations. Front Nutr. (2019) 6:127. doi: 10.3389/fnut.2019.00127 31475151 PMC6702319

[18] Mariotti F Tomé D Mirand PP. Converting nitrogen into protein - beyond 6.25 and Jones' factors. Crit Rev Food Sci Nutr. (2008) 48:177–84. doi: 10.1080/10408390701279749 18274971

[19] Saldanha do Carmo C Silventoinen-Veijalainen P Zobel H Holopainen-Mantila U Sahlstrøm S Knutsen SH. The effect of dehulling of yellow peas and faba beans on the distribution of carbohydrates upon dry fractionation. LWT. (2022) 163:113509. doi: 10.1016/j.lwt.2022.113509

[20] Prosky L Asp NG Furda I deVries JW Schweizer TF Harland BF . Determination of total dietary fiber in foods and food products: collaborative study. J AOAC Int. (1985) 68:677–9. doi: 10.1093/jaoac/68.4.677 2993226

[21] McCleary BV De Vries JW Rader JI Cohen G Prosky L Mugford DC . Determination of total dietary fiber (CODEX Definition) by enzymatic-gravimetric method and liquid chromatography: collaborative study. J AOAC Int. (2010) 93:221–33. doi: 10.1094/CFW-56-6-0238 20334184

[22] McCleary BV. Total dietary fiber (CODEX definition) in foods and food ingredients by a rapid enzymatic-gravimetric method and liquid chromatography: collaborative study, first action 2017.16. J AOAC Int. (2019) 102:196–07. doi: 10.5740/jaoacint.18-0180 30107867

[23] Bunzel M Ralph J Marita JM Hatfield RD Steinhart H. Diferulates as structural components in soluble and insoluble cereal dietary fibre. J Agric Food Chem. (2001) 81:653–60. doi: 10.1002/jsfa.861

[24] Saeman JF Bubl JL Harris EE. Quantitative saccharification of wood and cellulose. Ind. Eng Chem Anal Ed. (1945) 17:35–7. doi: 10.1021/i560137a008

[25] De Ruiter GA Schols HA Voragen AGJ Rombouts FM. Carbohydrate analysis of water-soluble uronic acid-containing polysaccharides with high-performance anion-exchange chromatography using methanolysis combined with TFA hydrolysis is superior to four other methods. Anal Biochem. (1992) 207:176–85. doi: 10.1016/0003-2697(92)90520-H 1489092

[26] Wefers D Bunzel M. Characterization of dietary fiber polysaccharides from dehulled common buckwheat (*Fagopyrum esculentum*) seeds. Cereal Chem. (2015) 92:598–603. doi: 10.1094/CCHEM-03-15-0056-R

[27] Nunes FM Coimbra MA. Chemical characterization of the high molecular weight material extracted with hot water from green and roasted arabica coffee. J Agric Food Chem. (2001) 49:1773–82. doi: 10.1021/jf0012953 11308325

[28] Gniechwitz D Reichardt N Blaut M Steinhart H Bunzel M. Dietary fiber from coffee beverage: degradation by human fecal microbiota. J Agric Food Chem. (2007) 55:6989–96. doi: 10.1021/jf070646b 17658822

[29] Sweet DP Shapiro RH Albersheim P. Quantitative analysis by various g.l.c. response-factor theories for partially methylated and partially ethylated alditol acetates. Carbohydr Res. (1975) 40:217–25. doi: 10.1016/S0008-6215(00)82604-X

[30] Steck J Kaufhold L Bunzel M. Structural profiling of xyloglucans from food plants by high-performance anion-exchange chromatography with parallel pulsed amperometric and mass spectrometric detection. J Agric Food Chem. (2021) 69:8838–49. doi: 10.1021/acs.jafc.1c02967 34339210

[31] Blumenkrantz N Asboe-Hansen G. New method for quantitative determination of uronic acids. Anal Biochem. (1973) 54:484–9. doi: 10.1016/0003-2697(73)90377-1 4269305

[32] Müller-Maatsch J Caligiani A Tedeschi T Elst K Sforza S. Simple and validated quantitative ^1^H NMR method for the determination of methylation, acetylation, and feruloylation degree of pectin. J Agric Food Chem. (2014) 62:9081–7. doi: 10.1021/jf502679s 25137229

[33] Schmid V Trabert A Keller J Bunzel M Karbstein HP Emin MA . Functionalization of enzymatically treated apple pomace from juice production by extrusion processing. Foods. (2021) 10:485. doi: 10.3390/foods10030485 33668342 PMC7996331

[34] Leijdekkers AGM Sanders MG Schols HA Gruppen H. Characterizing plant cell wall derived oligosaccharides using hydrophilic interaction chromatography with mass spectrometry detection. J Chromatogr A. (2011) 1218:9227–35. doi: 10.1016/j.chroma.2011.10.068 22099219

[35] Robertson JA de Monredon Dysseler FD Guillon P Amado FR Thibault J-F. Hydration properties of dietary fibre and resistant starch: a European collaborative study. LWT Food Sci Technol. (2000) 33:72–9. doi: 10.1006/fstl.1999.0595

[36] Le Goff A Renard CMGC Bonnin E Thibault JF. Extraction, purification and chemical characterisation of xylogalacturonans from pea hulls. Carbohydr Polym. (2001) 45:325–34. doi: 10.1016/S0144-8617(00)00271-X

[37] Willför S Pranovich A Tamminen T Puls J Laine C Suurnäkki A . Carbohydrate analysis of plant materials with uronic acid-containing polysaccharides - a comparison between different hydrolysis and subsequent chromatographic analytical techniques. Ind Crops Prod. (2009) 29:571–80. doi: 10.1016/j.indcrop.2008.11.003

[38] Fry SC. The structure and functions of xyloglucan. J Exp Bot. (1989) 40:1–11. doi: 10.1093/jxb/40.1.1

[39] Ralet M-C Saulnier L Thibault JF. Raw and extruded fibre from pea hulls. Part II: structural study of the water-soluble polysaccharides. Carbohydr Polym. (1993) 20:25–34. doi: 10.1016/0144-8617(93)90029-4

[40] Broxterman SE Picouet P Schols HA. Acetylated pectins in raw and heat processed carrots. Carbohydr Polym. (2017) 177:58–66. doi: 10.1016/j.carbpol.2017.08.118 28962796

[41] Renard CMGC Weightman RM Thibault JF. The xylose-rich pectins from pea hulls. Int J Biol Macromol. (1997) 21:155–62. doi: 10.1016/S0141-8130(97)00055-X 9283030

[42] Schmidt RE Bunzel M. Evaluation of AOAC-Method 2017.16: detection of oligosaccharides as low molecular weight soluble dietary fiber. Cereal Chem. (2025) 102:431–7. doi: 10.1002/cche.10885

[43] Nieto-Dominguez M de Eugenio LI York-Duran MJ Rodriguez-Colinas B Plou FJ Chenoll E . Prebiotic effect of xylooligosaccharides produced from birchwood xylan by a novel fungal GH11 xylanase. Food Chem. (2017) 232:105–13. doi: 10.1016/j.foodchem.2017.03.149 28490053

[44] Liu X Hu KKY Haritos VS. Enzymatic production of cello-oligosaccharides with potential human prebiotic activity and release of polyphenols from grape marc. Food Chem. (2024) 435:137562. doi: 10.1016/j.foodchem.2023.137562 37778264

[45] Fry SC. Plant cell walls. ELS. (2001) 1–11. doi: 10.1038/npg.els.0001682

[46] Chinga-Carrasco G. Cellulose fibres, nanofibrils and microfibrils: the morphological sequence of MFC components from a plant physiology and fibre technology point of view. Nanoscale Res Lett. (2011) 6:417. doi: 10.1186/1556-276X-6-417 21711944 PMC3211513

[47] Martínez-Monteagudo SI Yan B Balasubramaniam VM. Engineering process characterization of high-pressure homogenization - from laboratory to industrial scale. Food Eng Rev. (2017) 9:143–69. doi: 10.1007/s12393-016-9151-5

[48] Tosh SM Yada S. Dietary fibres in pulse seeds and fractions: characterization, functional attributes, and applications. Food Res Int. (2010) 43:450–60. doi: 10.1016/j.foodres.2009.09.005

[49] Taipale T Österberg M Nykänen A Ruokolainen J Laine J. Effect of microfibrillated cellulose and fines on the drainage of kraft pulp suspension and paper strength. Cellulose. (2010) 17:1005–20. doi: 10.1007/s10570-010-9431-9

[50] Déléris I Wallecan J. Relationship between processing history and functionality recovery after rehydration of dried cellulose-based suspensions: a critical review. Adv Colloid Interface Sci. (2017) 246:1–12. doi: 10.1016/j.cis.2017.06.013 28688780

[51] Agoda-Tandjawa G Durand S Berot S Blassel C Gaillard C Garnier C . Rheological characterization of microfibrillated cellulose suspensions after freezing. Carbohydr Polym. (2010) 80:677–86. doi: 10.1016/j.carbpol.2009.11.045

[52] Saarikoski E Saarinen T Salmela J Seppälä J. Flocculated flow of microfibrillated cellulose water suspensions: an imaging approach for characterisation of rheological behaviour. Cellulose. (2012) 19:647–59. doi: 10.1007/s10570-012-9661-0

[53] Chan SY Choo WS Young DJ Loh XJ. Pectin as a rheology modifier: origin, structure, commercial production and rheology. Carbohydr Polym. (2017) 161:118–39. doi: 10.1016/j.carbpol.2016.12.033 28189220

[54] Koponen AI. The effect of consistency on the shear rheology of aqueous suspensions of cellulose micro- and nanofibrils: a review. Cellulose. (2020) 27:1879–97. doi: 10.1007/s10570-019-02908-w

