# Supplementary material for: Production and characterization of mechanically-enzymatically treated suspensions of the food by-product pea hull
Source: Front Nutr. 2026 Jul 10;13:1852207. doi: 10.3389/fnut.2026.1852207 (PMC13398759; doi:10.3389/fnut.2026.1852207)
Supplement: Supplementary file 1 [file Supplementary_file_1.pdf]

Supplementary Material

Production and characterization of mechanically-enzymatically treated suspensions of the food by-product pea hull

Rebekka Elke Schmidt[1], Veronika Kurz[1], Verena Haitz[1], Rocío Morales-Medina[2], Jan Steffan[1], Judith Keller[1], Stephan Drusch[2], Mirko Bunzel[1*]

[1]Institute of Applied Biosciences, Department of Food Chemistry and Phytochemistry, Karlsruhe Institute of Technology (KIT), Karlsruhe, Germany

[2]Institute of Food Technology and Food Chemistry, Department of Food Technology and Food Material Science, Technische Universität Berlin (TUB), Berlin, Germany

*** Correspondence:**
Corresponding Author: mirko.bunzel@kit.edu

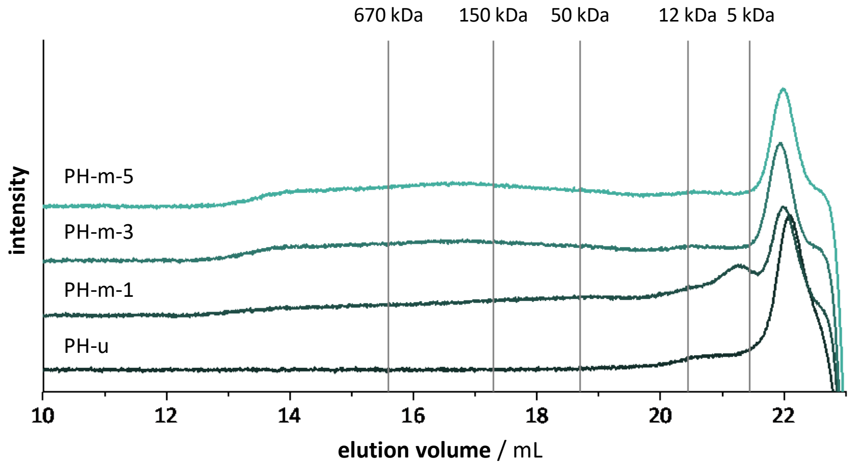

Fig. S1: Molecular weight distribution of soluble dietary fiber of untreated (PH-u) and mechanically treated pea hulls (PH-m-1/3/5) in reference to dextran standards (5-670 kDa) as molecular weight marker (n = 2; analytical replicates)

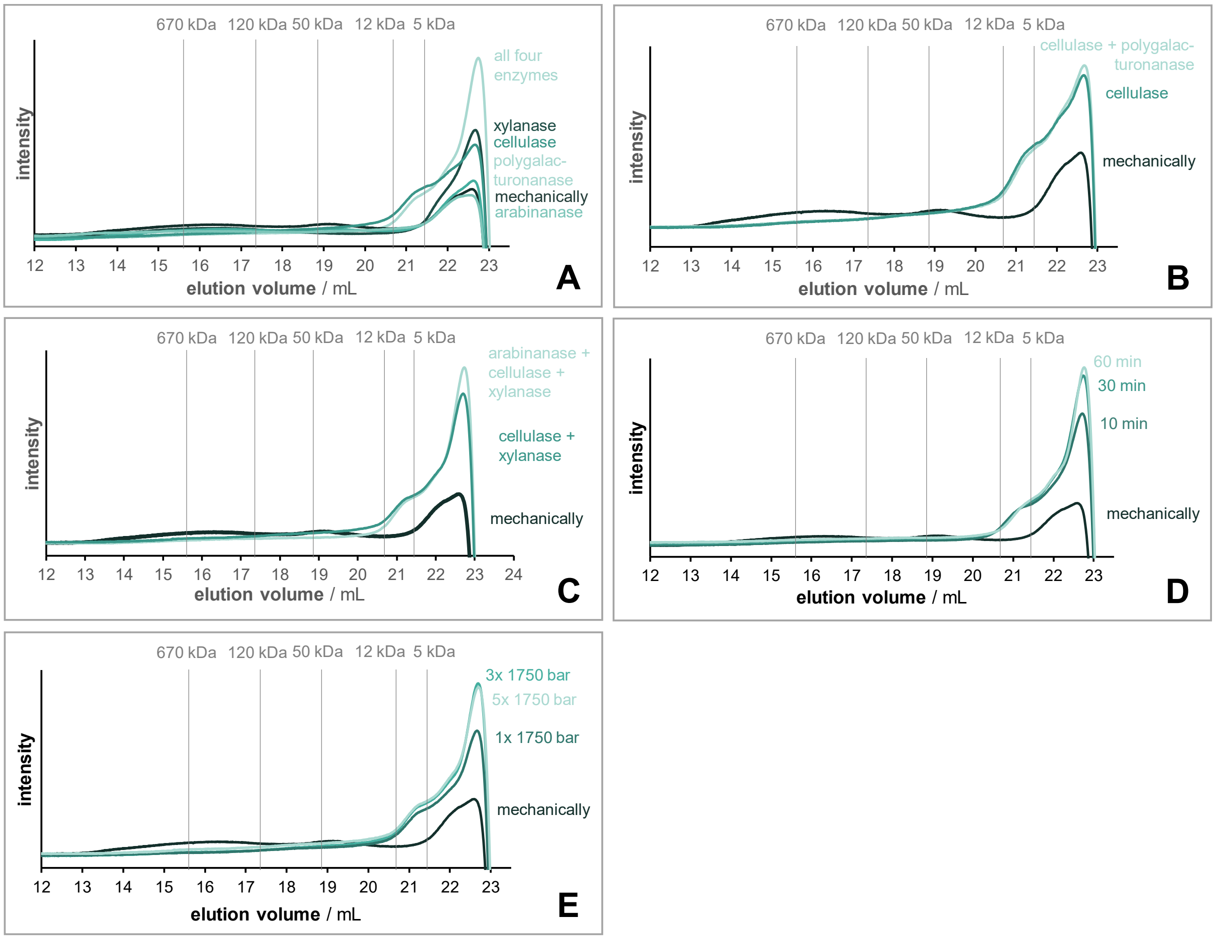

Fig. S2: Molar distribution of supernatants of different mechanically-enzymatically treated pea hull suspensions in water compared to mechanically treated pea hull suspension (5x 1750 bar) referred to dextran standards (5-670 kDa) as molecular weight marker. The enzymatic hydrolysis was done with (A) arabinanase, cellulase, polygalacturonanase, xylanase and a mixture of these four enzymes, with (B) cellulase or cellulase and polygalacturonanase, with (C) cellulase and xylanase with and without arabinanase, with (D) arabinanase, cellulase, polygalacturonanase, and xylanase for 10, 30, or 60 min, and with (E) cellulase and xylanase of various mechanically treated pea hulls (1x/3x/ 5x 1750 bar) Unless otherwise stated, the enzymatic treatment of mechanically treated pea hull (5x 1750 bar) was performed with an enzyme:substrate ratio of each enzyme of 1:100 at 50 °C for 30 min.

Tab. S1: Gradient program for the analysis of monosaccharides using high performance anion exchange chromatography (PA20 column) with pulsed amperometric detection and ultra-pure water (A), 0.1 M sodium hydroxide (B), and 0.1 M sodium hydroxide with 0.5 M sodium acetate (C) as eluents

time / min	A / %	B / %	C / %
-20.0	0	100	0
-10.0	0	100	0
-9.9	90	10	0
0.0	90	10	0
1.5	97	3	0
22.0	97	3	0
27.0	0	100	0
27.1	0	60	40
37.0	0	60	40

Tab. S2: Gradient program for analysis of oligosaccharides of the raffinose family using high performance anion exchange chromatography (PA20 column) with pulsed amperometric detection and ultra-pure water (A), 0.1 M sodium hydroxide (B), and 0.1 M sodium hydroxide with 0.5 M sodium acetate (C) as eluents

time / min	A / %	B / %	C / %
-20.0	0	100	0
-10.0	0	100	0
-9.9	80	20	0
0.0	80	20	0
1.5	97	3	0
22.0	97	3	0
32.0	0	100	0
37.0	0	100	0
37.1	0	60	40
47.0	0	60	40

Tab. S3: Gradient program for analysis of disaccharides (arabinobiose, cellobiose, and xylobiose) using high performance anion exchange chromatography (PA20 column) with pulsed amperometric detection and ultra-pure water (A), 0.1 M sodium hydroxide (B), and 0.1 M sodium hydroxide with 0.5 M sodium acetate (C) as eluents

time / min	A / %	B / %	C / %
-30.0	0	0	100
-20.0	0	0	100
-19.9	90	10	0
0.0	90	10	0
1.5	97	3	0
12.0	97	3	0
15.0	90	10	0
30.0	80	20	0
35.0	40	60	0
36.0	33	60	7
46.0	33	60	7
46.1	0	0	100
56.0	0	0	100

Tab. S4: Gradient program for analysis of digalacturonic acid using high performance anion exchange chromatography (PA20 column) with pulsed amperometric detection and ultra-pure water (A), 0.1 M sodium hydroxide (B), and 0.1 M sodium hydroxide with 0.5 M sodium acetate (C) as eluents

time / min	A / %	B / %	C / %
-30.0	0	0	100
-20.0	0	0	100
-19.9	90	10	0
0.1	90	10	0
10.0	50	50	0
20.0	0	80	20
29.0	0	56	44
29.1	0	0	100
40.0	0	0	100

Tab. S5: Monomer composition of the polysaccharides released from insoluble (IDF) and soluble dietary fiber (SDF) of pea hull after hydrolysis with sulfuric acid and/or methanolysis and trifluoroacetic acid (TFA)-hydrolysis in mol% ± range/2 (n = 2; analytical replicates).

monosaccharide	molar proportion / mol%		
	**IDF** sulfuric acid hydrolysis	**IDF** methanolysis + TFA-hydrolysis	**SDF** methanolysis + TFA-hydrolysis
glucuronic acid	-	0.79 ± 0.00	1.73 ± 0.04
galacturonic acid	6.66 ± 0.32	22.85 ± 0.01	16.71 ± 2.07
mannose	-	-	3.98 ± 0.05
xylose	18.13 ± 0.29	30.50 ± 0.30	15.33 ± 0.24
glucose	64.30 ± 0.49	4.91 ± 0.47	0.96 ± 0.00
galactose	2.00 ± 0.09	7.07 ± 0.25	9.88 ± 0.36
arabinose	7.29 ± 0.30	24.00 ± 0.37	43.58 ± 1.22
rhamnose	1.62 ± 0.06	8.08 ± 0.09	7.27 ± 0.71
fucose	-	1.80 ± 0.04	0.56 ± 0.04

Tab. S6: Results of xyloglucan profiling of insoluble dietary fiber from pea hull: Molar distribution (mol%) of enzymatically released xyloglucan oligosaccharides according to the nomenclature of Fry et al. (1993) and molar monomer composition of xyloglucan oligosaccharides (mol%) ± range/2 (n = 2; analytical replicates)

xyloglucan-oligosaccharide	molar distribution / mol%	monosaccharide	molar distribution / mol%
XG	19.7 ± 0.7	glucose	50.5 ± 0.1
XXG	1.6 ± 0.0		
XXXG	2.6 ± 0.5	xylose	37.0 ± 0.1
XX	21.8 ± 0.8		
XXX	< LOQ	galactose	7.0 ± 0.1
XLG	0.8 ± 0.0		
XXLG	5.0 ± 0.2	fucose	5.4 ± 0.1
XFG	4.6 ± 0.4		
XXFG	35.6 ± 1.1		
XLFG	8.1 ± 0.2		

Tab. S7: Weight amount (% of dried pea hull) of dietary fiber classified as insoluble (IDF), soluble (SDF), and low-molecular weight soluble dietary fiber (LMWSDF) of untreated (PH-u) and mechanically treated pea hulls (PH-m-1/3/5) ± standard deviation (n = 3; analytical replicates)

	**weight amount** / %			
**dietary fiber**	**PH-u**	**PH-m-1**	**PH-m-3**	**PH-m-5**
IDF	70.4 ± 0.9	76.0 ± 0.4	73.4 ± 0.2	72.5 ± 0.1
SDF	5.3 ± 1.0	4.9 ± 0.1	6.0 ± 0.1	7.0 ± 0.0
LMWSDF	0.6 ± 0.0	0.9 ± 0.2	0.9 ± 0.1	0.9 ± 0.1

Tab. S8: Monomer composition of the polysaccharides released from insoluble (IDF) and soluble dietary fiber (SDF) of untreated (PH-u) and mechanically treated pea hull (PH-m-1/3/5) after hydrolysis with sulfuric acid and/or methanolysis and trifluoro acetic acid (TFA)-hydrolysis in mol% ± range/2 (n = 2; analytical replicates).

	**molar proportion** / mol%			
**monosaccharide**	**PH-u**	**PH-m-1**	**PH-m-3**	**PH-m-5**
	**IDF** sulfuric acid hydrolysis			
galacturonic acid	6.66 ± 0.32	7.27 ± 0.22	5.33 ± 0.02	4.99 ± 0.03
xylose	18.13 ± 0.29	13.52 ± 0.11	14.17 ± 0.01	13.77 ± 0.00
glucose	64.30 ± 0.49	73.20 ± 0.05	74.51 ± 0.00	75.80 ± 0.00
galactose	2.00 ± 0.09	1.04 ± 0.06	0.99 ± 0.01	0.93 ± 0.01
arabinose	7.29 ± 0.30	3.76 ± 0.07	3.80 ± 0.01	3.40 ± 0.01
rhamnose	1.62 ± 0.06	1.23 ± 0.06	1.21 ± 0.00	1.11 ± 0.01
	**IDF** methanolysis + TFA-hydrolysis			
glucuronic acid	0.79 ± 0.00	-	0.48 ± 0.09	-
galacturonic acid	22.85 ± 0.01	23.02 ± 0.01	26.52 ± 0.00	22.66 ± 0.02
xylose	30.50 ± 0.30	37.40 ± 0.01	35.72 ± 0.01	39.72 ± 0.03
glucose	4.91 ± 0.47	7.26 ± 0.02	7.64 ± 0.03	6.93 ± 0.18
galactose	7.07 ± 0.25	4.80 ± 0.01	4.19 ± 0.02	4.47 ± 0.02
arabinose	24.00 ± 0.37	19.17 ± 0.01	18.13 ± 0.00	18.45 ± 0.02
rhamnose	8.08 ± 0.09	6.90 ± 0.00	5.89 ± 0.01	6.26 ± 0.00
fucose	1.80 ± 0.04	1.45 ± 0.01	1.43 ± 0.02	1.50 ± 0.03
	**SDF** methanolysis + TFA-hydrolysis			
glucuronic acid	1.73 ± 0.04	0.85 ± 0.29	-	1.07 ± 0.01
galacturonic acid	16.71 ± 2.07	22.33 ± 0.21	14.82 ± 0.01	21.92 ± 0.03
mannose	3.98 ± 0.05	4.49 ± 0.02	4.06 ± 0.00	2.58 ± 0.01
xylose	15.33 ± 0.24	16.88 ± 0.06	12.96 ± 0.00	12.37 ± 0.02
glucose	0.96 ± 0.00	1.45 ± 0.11	-	0.66 ± 0.07
galactose	9.88 ± 0.36	8.41 ± 0.07	11.54 ± 0.01	9.56 ± 0.01
arabinose	43.58 ± 1.22	36.44 ± 0.08	42.75 ± 0.00	42.74 ± 0.02
rhamnose	7.27 ± 0.71	9.14 ± 0.00	13.87 ± 0.01	8.54 ± 0.02
fucose	0.56 ± 0.04	-	-	0.55 ± 0.03

Tab. S9: Molar distribution (mol%) and total amount (µmol/g dried pea hull) of enzymatically released arabinooligosaccharides of insoluble (IDF) and soluble dietary fiber (SDF) from untreated (PH-u) and mechanically treated pea hull (PH-m-1/3/5) as analyzed by the arabinan profiling approach in mol% ± range/2 (n = 2; analytical replicates); A2a, A4a, … = nomenclature of arabinooligosaccharides according to Wefers and Bunzel (2016); LOQ = limit of quantification

	**PH-u**	**PH-m-1**	**PH-m-3**	**PH-m-5**
		**IDF**		
		**molar distribution** / mol%		
**A2a**	84.6 ± 0.0	87.0 ± 0.0	89.6 ± 0.0	90.7 ± 0.0
**A4a**	1.1 ± 0.0	1.0 ± 0.0	0.9 ± 0.0	0.8 ± 0.0
**A4b**	12.0 ± 0.0	10.2 ± 0.0	8.2 ± 0.0	7.2 ± 0.0
**A5a**	< LOQ	< LOQ	< LOQ	< LOQ
**A7b**	2.3 ± 0.0	1.8 ± 0.0	1.4 ± 0.0	1.2 ± 0.0
		**absolute amount** / µmol/g		
	14.1 ± 1.2	10.7 ± 0.1	12.7 ± 0.2	13.3 ± 0.3
		**SDF**		
		**molar distribution** / mol%		
**A2a**	84.9 ± 0.3	92.6 ± 0.1	91.2 ± 0.2	90.7 ± 0.6
**A4a**	1.7 ± 0.0	< LOQ	< LOQ	1.3 ± 0.0
**A4b**	9.7 ± 0.2	6.2 ± 0.1	7.3 ± 0.1	6.7 ± 0.5
**A5a**	0.5 ± 0.0	-	-	-
**A7a**	1.5 ± 0.0	-	-	-
**A7b**	1.7 ± 0.1	1.2 ± 0.0	1.5 ± 0.1	1.3 ± 0.1
		**absolute amount** / µmol/g		
	311.1 ± 14.1	107.6 ± 0.1	77.9 ± 7.5	108.8 ± 7.8

Tab. S10: Weight amount (% of dried pea hull) of dietary fiber classified as insoluble (IDF), soluble (SDF), and low-molecular weight soluble dietary fiber (LMWSDF) and of free mono- and disaccharides of the untreated (PH-u), mechanically (PH-m-3/PH-m-5), and mechanically-enzymatically treated pea hulls (PH-me-3/PH-me-5) ± range/2 (n = 2; analytical replicates)

	**weight amount / %**				
	**PH-u**	**PH-m-3**	**PH-me-3**	**PH-m-5**	**PH-me-5**
**dietary fiber composition**					
IDF	70.4 ± 0.9	73.4 ± 0.2	61.2 ± 1.0	72.5 ± 0.1	61.6 ± 0.3
SDF	5.3 ± 1.0	6.0 ± 0.1	7.3 ± 0.9	7.0 ± 0.0	7.2 ± 1.2
LMWSDF	0.6 ± 0.0	0.9 ± 0.1	6.0 ± 1.4	0.9 ± 0.1	3.9 ± 0.4
**free mono- and disaccharides**					
digalacturonic acid	-	-	0.37 ± 0.05	-	0.25 ± 0.01
galacturonic acid	-	0.05 ± 0.00	0.25 ± 0.01	0.05 ± 0.00	0.26 ± 0.01
xylobiose	-	-	0.92 ± 0.01	-	1.09 ± 0.01
xylose	-	-	0.03 ± 0.00	-	0.03 ± 0.00
cellobiose	-	-	1.16 ± 0.02	-	1.58 ± 0.02
glucose	0.03 ± 0.00	0.16 ± 0.00	1.00 ± 0.01	0.16 ± 0.00	1.33 ± 0.00
arabinobiose	-	-	1.04 ± 0.02	-	1.49 ± 0.01
arabinose	-	0.01 ± 0.00	0.37 ± 0.00	0.01 ± 0.00	0.44 ± 0.00
fructose	0.01 ± 0.00	0.11 ± 0.00	0.12 ± 0.01	0.11 ± 0.00	0.14 ± 0.00
galactose	-	0.03 ± 0.00	0.09 ± 0.00	0.03 ± 0.00	0.10 ± 0.00
rhamnose	-	-	0.04 ± 0.00	-	0.05 ± 0.00

Tab. S11: Monosaccharide composition (mol%) after methanolysis and trifluoroacetic acid hydrolysis of insoluble (IDF), soluble (SDF), and low-molecular weight soluble dietary fiber (LMWSDF)-fractions of untreated (PH-u), mechanically (PH-m-3/PH-m-5), and mechanically-enzymatically treated pea hulls (PH-me-3/PH-me-5) ± range/2 (n = 2; analytical replicates)

	**molar proportion** / mol%				
**monosaccharide**	**PH-u**	**PH-m-3**	**PH-me-3**	**PH-m-5**	**PH-me-5**
			**IDF**		
glucuronic acid	0.79 ± 0.00	0,48 ± 0.09	0.59 ± 0.05	-	0.48 ± 0.02
galacturonic acid	22.85 ± 0.01	26.52 ± 0.00	28.51 ± 0.83	22.66 ± 0.02	27.13 ± 0.23
xylose	30.50 ± 0.30	35.72 ± 0.01	32.07 ± 0.47	39.72 ± 0.03	34.73 ± 1.45
glucose	4.91 ± 0.47	7.64 ± 0.03	19.64 ± 0.73	6.93 ± 0.18	13.50 ± 2.01
galactose	7.07 ± 0.25	4.19 ± 0.02	4.86 ± 0.11	4.47 ± 0.02	5.41 ± 0.27
arabinose	24.00 ± 0.37	18.13 ± 0.00	8.93 ± 0.04	18.45 ± 0.02	12.47 ± 0.40
rhamnose	8.08 ± 0.09	5.89 ± 0.01	2.73 ± 0.15	6.26 ± 0.00	3.32 ± 0.05
fucose	1.80 ± 0.04	1.43 ± 0.02	2.67 ± 0.01	1.50 ± 0.03	2.96 ± 0.08
			**SDF**		
glucuronic acid	1.73 ± 0.04	-	1.46 ± 0.03	1.07 ± 0.01	1.41 ± 0.01
galacturonic acid	16.71 ± 2.07	14.82 ± 0.01	32.04 ± 0.34	21.92 ± 0.03	30.21 ± 0.33
mannose	3.98 ± 0.05	4.06 ± 0.00	3.75 ± 0.00	2.58 ± 0.01	3.83 ± 0.08
xylose	15.33 ± 0.24	12.96 ± 0.00	10.09 ± 0.02	12.37 ± 0.02	9.17 ± 0.10
glucose	0.96 ± 0.00	-	1.02 ± 0.00	0.66 ± 0.07	1.04 ± 0.00
galactose	9.88 ± 0.36	11.54 ± 0.01	14.61 ± 0.01	9.56 ± 0.01	14.55 ± 0.02
arabinose	43.58 ± 1.22	42.75 ± 0.00	13.22 ± 0.04	42.74 ± 0.02	16.02 ± 0.11
rhamnose	7.27 ± 0.71	13.87 ± 0.01	22.70 ± 0.31	8.54 ± 0.02	22.66 ± 0.38
fucose	0.56 ± 0.04	-	1.11 ± 0.01	0.55 ± 0.03	1.10 ± 0.00
			**LMWSDF-fraction**		
glucuronic acid	2.59 ± 0.15	2.04 ± 0.03	0.87 ± 0.00	1.97 ± 0.09	0.78 ± 0.05
galacturonic acid	5.09 ± 0.36	2.26 ± 0.37	5.59 ± 0.17	2.42 ± 0.28	5.31 ± 0.52
xylose	10.41 ± 1.13	4.84 ± 1.95	18.70 ± 0.29	4.05 ± 0.29	20.56 ± 0.07
glucose	49.26 ± 1.95	69.18 ± 3.17	44.99 ± 0.61	70.87 ± 0.66	42.98 ± 0.50
galactose	21.33 ± 0.15	15.67 ± 0.19	5.10 ± 0.03	14.91 ± 0.09	4.22 ± 0.31
arabinose	9.07 ± 0.21	6.01 ± 0.62	21.18 ± 0.18	5.79 ± 0.10	23.20 ± 0.51
rhamnose	2.24 ± 0.04	-	3.57 ± 0.00	-	2.95 ± 0.19

Tab. S12: Monosaccharide composition (mol%) after sulfuric acid hydrolysis of insoluble dietary fiber of untreated (PH-u), mechanically (PH-m-3/5), and mechanically-enzymatically treated pea hulls (PH-me-3/5) ± range/2 (n = 2; analytical replicates)

	molar proportion / mol%				
**monosaccharide**	**PH-u**	**PH-m-3**	**PH-me-3**	**PH-m-5**	**PH-me-5**
galacturonic acid	6.66 ± 0.32	5.33 ± 0.02	4.19 ± 0.13	4.99 ± 0.03	3.81 ± 0.19
xylose	18.13 ± 0.29	14.17 ± 0.01	11.32 ± 0.31	13.77 ± 0.00	11.70 ± 0.33
glucose	64.30 ± 0.49	74.51 ± 0.00	84.49 ± 0.44	75.80 ± 0.00	84.49 ± 0.14
galactose	2.00 ± 0.09	0.99 ± 0.01	-	0.93 ± 0.01	-
arabinose	7.29 ± 0.30	3.80 ± 0.01	-	3.40 ± 0.01	-
rhamnose	1.62 ± 0.06	1.21 ± 0.00	-	1.11 ± 0.01	-

Tab. S13: Molar distribution (mol%) and total amount (µmol/g dried pea hull) of enzymatically released arabinooligosaccharides of insoluble (IDF), and soluble dietary fiber (SDF) from untreated (PH-u), mechanically (PH-m-3/PH-m-5), and mechanically-enzymatically treated pea hulls (PH-me-3/PH-me-5) during arabinan profiling ± range/2 (n = 2); A2a, A4a, … = nomenclature of arabinooligosaccharides according to Wefers and Bunzel (2016), LOQ = limit of quantification

	**PH-u**	**PH-m-3**	**PH-me-3**	**PH-m-5**	**PH-me-5**
**IDF**					
**molar distribution** / mol%					
**A2a**	84.6 ± 0.0	89.6 ± 0.0	92.5 ± 0.1	90.7 ± 0.0	92.7 ± 0.0
**A4a**	1.1 ± 0.0	0.9 ± 0.0	< LOQ	0.8 ± 0.0	< LOQ
**A4b**	12.0 ± 0.0	8.2 ± 0.0	7.5 ± 0.1	7.2 ± 0.0	7.3 ± 0.0
**A5a**	< LOQ	< LOQ	< LOQ	< LOQ	< LOQ
**A7b**	2.3 ± 0.0	1.4 ± 0.0	< LOQ	1.2 ± 0.0	< LOQ
**absolute amount** / µmol/g					
	14.1 ± 1.2	12.7 ± 0.2	1.6 ± 0.0	13.3 ± 0.3	1.4 ± 0.0
**SDF**					
**molar distribution** / mol%					
**A2a**	84.9 ± 0.3	91.2 ± 0.2	95.3 ± 0.1	90.7 ± 0.6	95.6 ± 0.0
**A4a**	1.7 ± 0.0	< LOQ	< LOQ	1.3 ± 0.0	< LOQ
**A4b**	9.7 ± 0.2	7.3 ± 0.1	4.7 ± 0.2	6.7 ± 0.5	4.4 ± 0.0
**A5a**	0.5 ± 0.0	-	-	-	-
**A7a**	1.5 ± 0.0	-	-	-	-
**A7b**	1.7 ± 0.1	1.5 ± 0.1	-	1.3 ± 0.1	-
**absolute amount** / µmol/g					
	311.1 ± 14.1	77.9 ± 7.5	59.7 ± 4.0	108.8 ± 7.8	42.5 ± 0.5

Tab. S14: Weight amount (% of dried pea hull) of quantifiable oligosaccharides in the low-molecular weight soluble dietary fiber-fractions of the mechanically-enzymatically treated pea hulls (PH-me-3/PH-me-5) ± range/2 (n = 2; analytical replicates); n.q. = non-quantifiable

oligosaccharide	**weight amount** / %<br>**PH-me-3**	 **PH-me-5**
arabinotriose	1.34 ± 0.00	1.46 ± 0.01
arabinotetraose	0.46 ± 0.00	0.52 ± 0.00
cellotriose	0.04 ± 0.00	0.04 ± 0.00
cellotetraose	0.05 ± 0.00	n.q.
xylotriose	0.63 ± 0.02	0.61 ± 0.04
xylotetraose	0.43 ± 0.01	0.43 ± 0.03
xylopentaose	0.16 ± 0.00	0.17 ± 0.01
xylohexaose	0.09 ± 0.00	0.09 ± 0.00

Tab. S15: Particle size (D_{50}/D_{90}) of untreated (PH-u), mechanically (PH-m-3/PH-m-5), and mechanically-enzymatically treated pea hulls (PH-me-3/PH-me-5) ± standard deviation (n = 6, two batches in analytical triplicates)

	**D_{50}**[1] / µm	**D_{90}**[1] / µm
**PH-u**	142.2 ± 4.4	242.7 ± 7.9
**PH-m-3**	46.9 ± 0.8	120.7 ± 4.5
**PH-m-5**	33.9 ± 4.6	93.7 ± 6.8
**PH-me-3**	55.0 ± 0.6	116.1 ± 2.5
**PH-me-5**	59.7 ± 3.3	105.8 ± 6.9

References:

Fry, S. C., York, W. S., Albersheim, P., Darvill, A., Hayashi, T., Joseleau, J.-P., et al. (1993). An unambiguous nomenclature for xyloglucan-derived oligosaccharides. Physiol. Plant., 89:1. https://doi.org/10.1111/j.1399-3054.1993.tb01778.x

Wefers, D., and Bunzel, M. (2016). Arabinan and galactan oligosaccharide profiling by high-performance anion-exchange chromatography with pulsed amperometric detection (HPAEC-PAD). J. Agric. Food Chem., 64:22. https://doi.org/10.1021/acs.jafc.6b01121
